# The Chromatin Modifier MSK1/2 Suppresses Endocrine Cell Fates during Mouse Pancreatic Development

**DOI:** 10.1371/journal.pone.0166703

**Published:** 2016-12-14

**Authors:** Neha Bhat, Jeehye Park, Huda Y. Zoghbi, J. Simon C. Arthur, Kenneth S. Zaret

**Affiliations:** 1 Institute for Regenerative Medicine, Department of Cell and Developmental Biology, Perelman School of Medicine, University of Pennsylvania, Civic Center Blvd., Philadelphia, PA, United States of America; 2 Howard Hughes Medical Institute, Department of Molecular and Human Genetics, Baylor College of Medicine, and Jan and Dan Duncan Neurological Research Institute at Texas Children’s Hospital, Houston, TX, United States of America; 3 Medical Research Council Protein Phosphorylation Unit, School of Life Sciences, University of Dundee, Dundee, United Kingdom; Vrije Universiteit Brussel, BELGIUM

## Abstract

Type I diabetes is caused by loss of insulin-secreting beta cells. To identify novel, pharmacologically-targetable histone-modifying proteins that enhance beta cell production from pancreatic progenitors, we performed a screen for histone modifications induced by signal transduction pathways at key pancreatic genes. The screen led us to investigate the temporal dynamics of ser-28 phosphorylated histone H3 (H3S28ph) and its upstream kinases, MSK1 and MSK2 (MSK1/2). H3S28ph and MSK1/2 were enriched at the key endocrine and acinar promoters in E12.5 multipotent pancreatic progenitors. Pharmacological inhibition of MSK1/2 in embryonic pancreatic explants promoted the specification of endocrine fates, including the beta-cell lineage, while depleting acinar fates. Germline knockout of both *Msk* isoforms caused enhancement of alpha cells and a reduction in acinar differentiation, while monoallelic loss of *Msk1* promoted beta cell mass. Our screen of chromatin state dynamics can be applied to other developmental contexts to reveal new pathways and approaches to modulate cell fates.

## Introduction

Cell fate specification during development involves activation and repression of specific gene-regulatory networks, which are driven by changes in extracellular environment. Dynamics in such networks are mediated by lineage-specific transcription factors that recruit, among other proteins, histone-modifying enzymes to relevant loci [1, 2]. In many instances, the histone-modifying enzymes are themselves regulated by changes in the extracellular environment, thus mediating responses to inductive cues [3–6]. The loss of function or pharmacological inhibition of histone modifying enzymes in the progenitors of various lineages has been shown to modulate their eventual fate choice [7, 8]. We examined histone modifications induced by signaling pathways in the pancreatic beta cell progenitors, focusing on genes that are key drivers of different pancreatic lineages, with the aim of identifying pharmacologically-targetable histone modifiers that could promote pancreatic beta cell development.

MSK1/2 (Mitogen and Stress-induced Kinase) are partially redundant serine/threonine kinases that are phosphorylated downstream of MAPK (Erk and/or p38-mediated) and cAMP signal transduction pathways. Phosphorylated-MSK1/2 can, in turn, directly phosphorylate histone H3 at Ser10 and Ser28 residues, leading to transcriptional activation [9–18]. Yet recent reports also indicate the association of H3S10ph and H3S28ph with transcriptionally silent genes, suggesting context-dependent association of these modifications with the transcriptional status of a gene [19, 20]. The dynamics of MSK1/2-mediated H3S28 and H3S10 phosphorylation have been well characterized in signal-mediated transcriptional regulation in mammalian fibroblasts and Drosophila salivary glands (see references above), but their role has not been examined during pancreatic development in any metazoan.

Mouse pancreatic development begins with the specification of multipotent progenitors that co-express transcription factors *Pdx1*, *Ptf1a*, *Cpa1*, *Oc1*, and *Sox9* from 8.5 to 12.5 embryonic days of gestation (E8.5 to E12.5) [21–27]. During subsequent morphogenesis, proacinar exocrine precursors are restricted to the tips of branching pancreatic epithelia co-expressing *Ptf1a*, *Cpa1*, *Nr5a2*, *Gata4*, and *Mist1*. These genes orchestrate steps related to acinar differentiation, morphogenesis, and growth [27–32]. *Sox9* and *Nkx6*.*1* are expressed in the branching trunk domain, containing progenitors for duct and endocrine cell lineages [27, 33–35]. *Oc1* and *Pdx1* are co-expressed in the acinar precursors until E16.5. Subsequently, their expression diminishes in the acinar cells and increases in the duct and Insulin1/2 positive beta cell lineages, respectively, in the neonates [36–42].

All pancreatic endocrine cell types, including the beta cell lineage, are specified by the transcription factor *Neurogenin3* (*Neurog3*), which in turn activates other endocrine progenitor genes, such as *Pax6*, *NeuroD1*, *Insm1*, and *Rfx6* [43–48]. These endocrine progenitors differentiate into glucagon (Gcg)-producing alpha cells throughout pancreatic development, while the majority of mature insulin1 (Ins1/2) producing beta cells arise only after E13.5 [49–51]. The mature beta cells subsequently express high levels of *MafA*, *Glut2*, and *Pcsk1* [52,53].

In this study, we sought to identify novel regulators of beta cell specification, focusing on histone modifying enzymes that were previously identified as effectors of signal transduction pathways. To this end, we performed a screen for signaling-induced histone modifications at genes involved in the differentiation of pancreatic lineages. We found enrichment of H3S28ph, at the *Pdx1* promoter and at the *Pdx1* area II enhancer (*Pdx1*^*enh*^) in E12.5 multipotent pancreatic progenitors. The *Pdx1*^*enh*^ is necessary for differentiation of the beta cell lineage [54–60]. The *Ptf1a* gene, critical for acinar cell specification [27–29], and acinar-cell specific *Amylase2a (Amyl)* promoter were enriched for H3S28ph in E12.5 multipotent pancreatic progenitors. In pancreatic explants harvested from E12.5 and E15.5 stages of pancreatic development, pharmacological inhibition of Mitogen and Stress Activated Kinase (MSK1/2), an upstream chromatin modifier of H3S28ph and H3S10ph, using SB-747541A, caused a strong induction of the endocrine fates, including the beta-cell lineage, while suppressing acinar fates. Germline knockouts of both *Msk1* and *Msk2* led to a decrease in acinar mass with an increase in alpha cell mass, consistent with alpha cells being the preferentially specified endocrine fate early in pancreatic development [51]. In accord with the robust induction of beta cell mass upon SB-747541A treatment at E15.5, monoallelic knockout of *Msk1* showed an enhancement of beta cell mass. Altogether, we find that the chromatin modifiers MSK1 and MSK2 normally promote acinar and suppress endocrine differentiation during pancreatic development, and that pharmacologic inhibition of MSK proteins can markedly enhance beta cell production at the later stage of endocrine differentiation. Our analysis of chromatin state dynamics can be applied to other developmental contexts to discover new ways to modulate cell fate decisions.

## Results

### A screen for histone modifications induced by signaling pathways

We predicted that performing a screen for signaling-induced histone modifications in pancreatic progenitors would reveal chromatin modifiers and signaling pathways that modulate beta cell induction, and that pharmacologic or genetic targeting of relevant modifiers could enhance beta cell production. To establish the screen, we considered Onecut1 (Oc1) as a potential marker to purify pancreatic epithelial cells, at E15.5, by Fluorescence Activated Cell Sorting (FACS). Using FACS, we separated the Oc1 positive cells, excluding Oc1 negative mesenchymal and maturing beta-cells. We verified by immunohistochemistry that only a minor 4.6% of total Ins1/2 positive cells were Oc1 positive at E15.5 stage of pancreatic development, consistent with other reports (S1A–S1C Fig, n = 3 pancreas, # of sections/pancreas = 5, [39–42]). Other lineage tracing studies have indicated that the Oc1+ trunk cells harbors progenitors of the mature beta-cell lineage [33–35]. Therefore, a screen for signaling induced histone modifications at key pancreatic lineage-specific genes in the Oc1+ epithelial progenitors could reveal signaling pathways that direct the induction of pancreatic progenitors towards the beta cell fates [1, 3–7].

**Fig 1:**
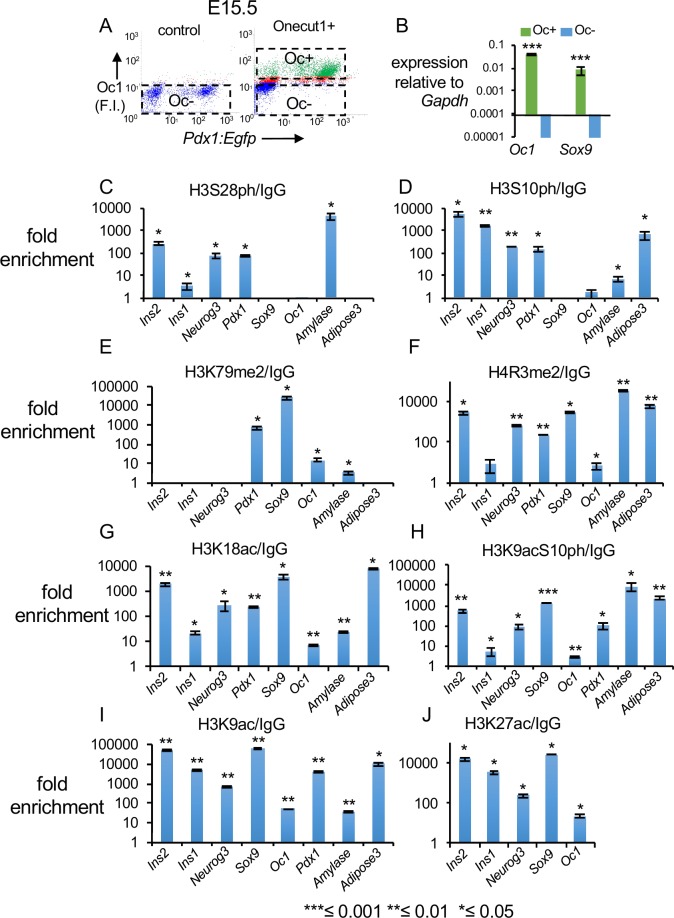
A screen for histone modifications down stream of signaling pathways in E15.5 pancreatic epithelial cells. (A) FACS scatter plots of cells treated with either IgG (control) or Onecut1 (Oc1+) antibody from *Pdx1*:*Egfp* E15.5 pancreata. The FACS purified Oc1+ cells, both Pdx1-GFP+ (Oc1+P+) and Pdx1-GFP- (Oc1+P-), were used for ChIP and qPCR (box labeled Oc+). The x and y axes indicate fluorescence intensity (F.I.). (B) RT-qPCR for ductal transcripts *Oc1* and *Sox9* in Oc+ (green) and Oc- (blue) cells. (C-J) Patterns for indicated histone modifications at select pancreatic genes (n = 3 technical replicates). *Ins2* (*Insulin2*), *Ins1* (*Insulin1*), *Neurog3* (*Neurogenin3*) and *Amyl* (*Amylase2a*). The y-axis shows enrichment over IgG. All P-values were calculated by Student’s t-test, one tailed.

For FACS sorting, a fluorophore-conjugated secondary antibody, lacking the Fc region, was used to stain and sort cells [61]. This step ensured that the chromatin bound by anti-Oc1 antibodies was not immunoprecipitated by Protein G agarose beads during a subsequent chromatin immunoprecipitation (ChIP) reaction. Also, the cells were fixed immediately after dissociation, prior to FACS, precluding any effects that may arise in the time after dissociation and sorting. The sort was done in *Pdx1*:*Egfp* [62] pancreata, enabling subsequent work, but we did not sort for Egfp in this screen (x-axis, Fig 1A). The Oc1+ population showed significantly higher expression of trunk and future duct genes *Oc1* and *Sox9* by RT-qPCR, indicating that we have enriched for Oc1+ cells by FACS (Fig 1A and 1B).

Next, we examined histone modifications induced by known signal transduction pathways (Table 1) by ChIP in the FACS-enriched Oc1+ population (Fig 1C–1J, n≥25 pooled pancreata for ChIP, n = 3 technical replicates). All P-values were calculated by a paired Student’s t test. H3S28ph was enriched at the promoters active in endocrine lineages, including *Insulin1 (Ins1*, P-value = 0.05), *Insulin2 (Ins2*, P-value = 0.05), *Neurog3* (P-value = 0.07), and the *Pdx1*^*enh*^ (P-value = 0.03), and also at the acinar specific *Amyl* promoter (P-value = 0.04, Fig 1C, Table 1). H3S10ph showed enrichment similar to H3S28ph at promoters for *Ins1* (P-value = 0.005), *Ins2* (P-value = 0.02), *Neurog3* (P-value = 0.002), *Pdx1*^*enh*^ (P-value = 0.04), and *Amyl*, (P-value = 0.03, Fig 1D, Table 1) genes. The gene promoters active in trunk and future duct cells, such as *Sox9* and *Oc1*, were neither enriched for H3S28ph nor H3S10ph (ChIP data summarized in Table 1). Conversely, H3K79me2 [63, 64] was significantly enriched at the promoters active in trunk and future duct epithelial cells, such as *Sox9* (P-value = 0.01), and *Oc1* (P-value = 0.01). The beta cell specific *Pdx1*^*enh*^ (P-value = 0.03), and acinar specific *Amyl* promoters were enriched for H3K79me2 as well (P-value = 0.05), Fig 1E, Table 1). H4R3me2 [65–67], H3K18ac [68], H3K9acS10ph, H3K9ac, and H3K27ac were significantly enriched at all the promoters examined and thus lacked specificity (Fig 1F–1J, Table 1). We focused on further investigating the dynamics of HS28ph and H3S10ph in purified E12.5 multipotent progenitors due to their enrichment at the endocrine and acinar specific promoters, and the relative lack at the ductal promoters, with the aim to identify chromatin modifiers that can modulate transcription from endocrine promoters.

**Table 1:** Summary of histone modifications, corresponding signaling pathways, and enrichment found in this study at gene promoters.

Histone Modifications	Signaling pathway	Promoters Positive	Promoters Negative
H3S28ph	• MAPK • Retinoic Acid • PI3K • TNF • Jak-Stat • cAMP/CREB [5, 9–20]	• *Ins1* • *Ins2* • *Pdx1* ^*enh*^ • *Neurog3* • *Amyl*	• *Onecut1* • *Sox9*
H3S10ph	• MAPK • Retinoic Acid • PI3K • TNF • Jak-Stat • cAMP/CREB [5,9–20]	• *Ins1* • *Ins2* • *Pdx1* ^*enh*^ • *Neurog3* • *Amyl*	• *Onecut1* • *Sox9*
H3K79me2	• Wnt • Retinoic Acid • Fgf [63, 64]	*Onecut1* • *Sox9* • *Pdx1* ^*enh*^ • *Amyl*	• *Ins1* • *Ins2* • *Neurog3*
H4R3me2	• BMP • Jak-Stat • Wnt • RA [65–67]	• *Ins1* • *Ins2* • *Pdx1*^*enh*^ • *Neurog3* • *Amyl* • *Sox9* • *Onecut1*	
H3K18ac	• PI3K [68]	• *Ins1* • *Ins2* • *Pdx1*^*enh*^ • *Neurog3* • *Amyl* • *Sox9* • *Onecut1*	

### Dynamics of H3S28ph and H3S10ph in E12.5 multipotent progenitors

**Fig 2:**
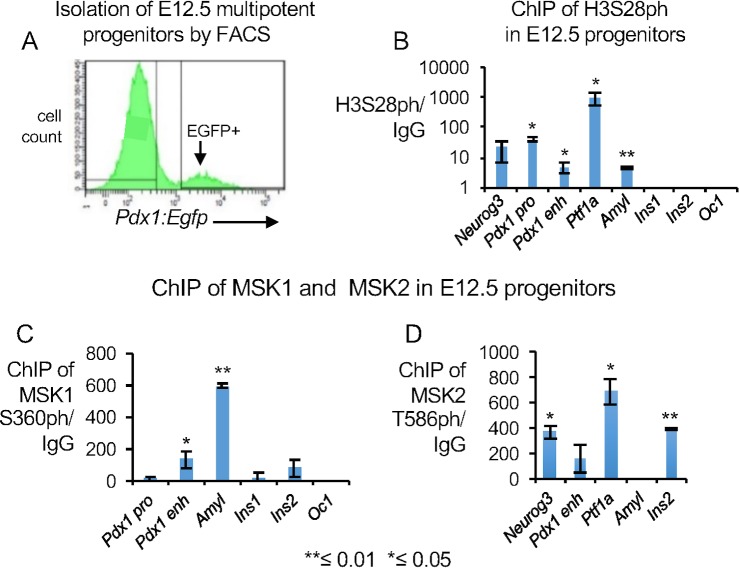
Dynamics of H3S28ph and the chromatin modifiers MSK1/2 in E12.5 FACS sorted cells. (A) Representative plot showing FACS sorted cells from E12.5 *Pdx1*: *Egfp* pancreata. (B) ChIP qPCR analysis for H3S28ph at select genes in E12.5 *Pdx1*: *Egfp* positive multipotent pancreatic progenitors, n≥150 pooled pancreata were used for each biological replicate. P-values for H3S28ph enrichment over IgG were: *Pdx1* promoter = 0.04, *Pdx1*^*enh*^ = 0.045, *Ptf1a* = 0.03 and *Amylase2a* = 0.01, n = 3 biological replicates and n = 3 technical replicates for H3S28ph. Values are averages of three independent experiments ±standard error. No significant enrichment could be found for H3S10ph at the select genes, with n = 2 biological replicates, n = 3 technical replicates of each biological replicate (data not shown). (C, D) ChIP-qPCR showing enrichment of MSK1S360ph over IgG (C) and MSK2T586ph over IgG (D) at the designated promoters in E12.5 multipotent pancreatic progenitors. P-values are 0.03, 0.007 respectively for *Pdx1*^enh^ and *Amyl* promoter in E12.5 pancreatic progenitors for MSK1ph and 0.04, 0.04 and 0.01 for *Neurog3*, *Ptf1a* and *Ins2* respectively for MSK2ph, n≥150 pooled pancreata for each biological replicate at E12.5. Values are averages of two independent experiments and 3 technical replicates ±standard error. Y-axis represents enrichment of H3S28ph, MSK1S360ph or MSK2T586ph over IgG. All P-values were calculated with a paired Student’s t test in independent experiments; *≤ 0.05, **≤ 0.01, one tailed.

We next examined H3S28ph and H3S10ph in FACS sorted Egfp+ multipotent pancreatic progenitors from pancreatic buds at an earlier E12.5 stage embryos of a *Pdx1*:*Egfp* genotype [62] (Figs 2A and S1D, n≥15 independent sorts). Since we dissected out the pancreatic buds, we excluded Egfp+ cells from stomach or duodenum [24] in addition to excluding the *Pdx1*:*Egfp* negative pancreatic mesenchymal cells by FACS [62], (Figs 2A and S1D). The isolated cells constitute a majority of the pancreatic epithelial cells at E12.5. RT-qPCR showed marked enrichment of the expected *Pdx1*, *Oc1*, *Cpa1*, *Sox9*, *Neurog3* and *Ptf1a* transcripts, while *Ins1*, *Ins2*, and *Amyl* were not expressed in the *Pdx1*:*Egfp+* population (S1E Fig), indicating the majority of the isolated cells are multipotent pancreatic epithelial progenitors. A total of 20,000 FACS purified *Pdx1*:*Egfp*+ cells, pooled from multiple sorts, were used both for antigen and IgG controls for each independent ChIP experiment.

H3S28ph showed significant enrichment at the *Pdx1* promoter and at the *Pdx1*^*enh*^ (Fig 2B, n≥150 pancreata were pooled for each ChIP experiment, n = 3 biological and n = 3 technical replicates for each experiment, P-value for *Pdx1* promoter = 0.04 and for *Pdx1*^*enh*^ = 0.045) in E12.5 multipotent progenitors. The *Ins1*, *Ins2*, *Neurog3* (endocrine) and *Oc1* (ductal) promoters did not show significant enrichment. The specific presence of H3S28ph at the *Pdx1*^*enh*^, previously implicated in beta-cell development [54–60], could reflect the activity of a chromatin modifier important for beta cell specification.

The promoters for the acinar lineage specific *Ptf1a* and *Amyl* genes were also significantly enriched for H3S28ph in E12.5 progenitors (Fig 2B, n≥150 pancreata were pooled for each independent ChIP experiment, n = 3 biological replicates and n = 3 technical replicates for each experiment, P-value for *Ptf1a* = 0.03 and for *Amyl* = 0.01). This is critical because *Ptf1a* is a key specifier of the acinar lineage [27–29] and *Amyl* is a definitive marker of acinar cell differentiation, and the specific enrichment at these promoters could represent an important step for modulating acinar differentiation. No gene promoters examined showed statistically significant enrichment for H3S10ph (n≥150 pooled pancreata for each biological replicate, n = 2 biological replicates, n = 3 technical replicates for each experiment; data not shown).

Altogether, the specific enrichment of H3S28ph at the endocrine and acinar lineage specific genes, but not ductal genes, in E12.5 multipotent pancreatic progenitors and in E15.5 Oc1+ pancreatic epithelial cells (Fig 1) suggests that these chromatin modifications could specifically modulate endocrine and acinar lineages during pancreatic development.

### Enrichment patterns of the chromatin modifiers of H3S28ph: MSK1/2 in E12.5 progenitors

Mitogen and Stress activated Kinases 1 and 2 (MSK/12) are the only known chromatin modifiers of H3S28ph and H3S10ph [9–20]. Hence, we examined whether phosphorylated MSK1 and MSK2 would directly bind at the endocrine and acinar lineage specific genes, relating to the enrichment of H3S28ph at both acinar and endocrine genes (Figs 1C and 2B). In E12.5 multipotent progenitors, we found significant enrichment of MSK1S360ph at the beta cell specific *Pdx1*^enh^, and enrichment of MSK2T586ph at the pan-endocrine *Neurog3* promoter (Fig 2C and 2D, n≥150 pooled pancreata for each biological replicate, n = 2 biological and 3 technical replicates, P-values are 0.03 and 0.04 respectively). Conversely, significant binding was detected at the *Amyl* promoter for MSK1S360ph and at the *Ptf1a* promoter for MSK2T586ph (Fig 2C and 2D, n≥150 pooled pancreata for each biological replicate, n = 2 biological and 3 technical replicates, P-values are 0.007 and 0.04 respectively). None of the other promoters examined showed significant binding. Altogether, these chromatin binding data suggest that MSK1 and MSK2 isoforms could directly modulate endocrine and acinar differentiation.

### Expression pattern of the MSK1 and MSK2 during pancreatic development

**Fig 3:**
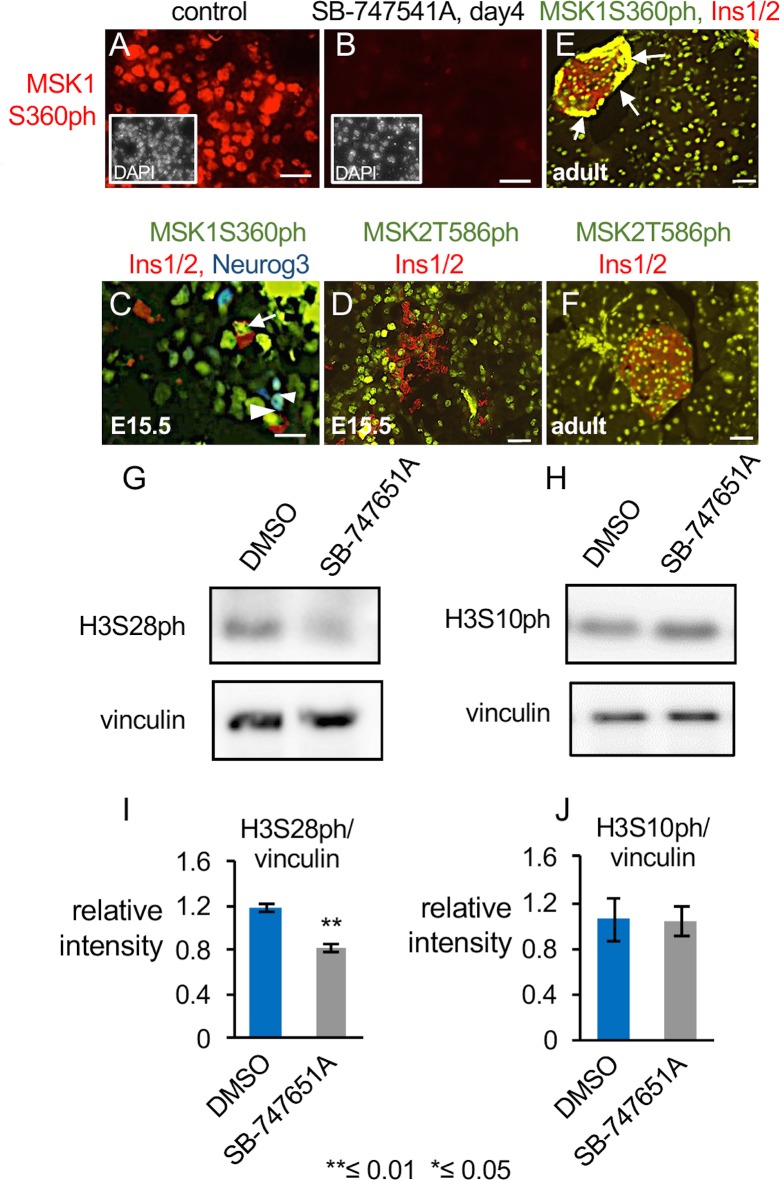
MSK1/2 is expressed in the pancreatic epithelium and mediates phosphorylation of Histone H3 at Ser28. (A, B) Epifluoroscent images showing expression of MSK1S360ph and DAPI (inset) in pancreatic sections from DMSO and SB-747541A treated explants at E15.5, cultured 4 days. DAPI stained images (inset) were pseudo-colored white to enhance contrast. n≥5 pancreata for each staining. Scale bar = 12.5 μm. (C-F) Expression of the indicated proteins in pancreatic sections from E15.5 stage (C, D) and (E, F) adult islets. Arrows in panel C indicate co-expression of MSK1S360ph with Ins1/2 (red), arrowheads indicate coexpression of MSK1S360ph with Neurog3 (blue). Arrows in panel E show high expression of MSK1S360ph at the exterior edge of the islet, possibly alpha cells. n≥5 pancreata for each staining, Scale bar for panel C = 12.5μm and for panel D-F = 50μm. (G, H) Western Blot for H3S28ph and H3S10ph in DMSO and SB-747541A treated explants, vinculin is the loading control, n = 2 biological replicates. (I, J) Quantification of the blots corresponding to panels G and H from two biological replicates.

MSK1Ser360ph (MSK1S360ph) and MSK2Thr586ph (MSK2T586ph) showed near ubiquitous expression in the nuclei of all pancreatic epithelial cells at E15.5 and in adult pancreata (Figs 3A and 3C–3F and S2A–S2D, n≥5 pancreata for each staining). The nuclear expression of MSK1/2 is consistent with their roles as chromatin modifiers (Fig 3A and 3C–3F). We further confirmed that sparsely present endocrine cells also expressed MSK1S360ph and MSK2Thr586ph. Out of all Ins1/2 positive cells counted, 74% of cells coexpressed MSK1S360ph (Fig 3C, arrows) and 87% of Neurog3 positive cells coexpressed MSK1S360ph (Fig 3C, arrowhead, n≥5 pancreata) at E15.5. Similarly, 75% of all Ins1/2+ cells co-expressed MSK2T586ph (Fig 3D, n≥5 pancreata) at E15.5. In the adult pancreata, both islets and exocrine tissues expressed MSK1S360ph and MSK2T586ph (Fig 3E and 3F), with considerably higher levels of MSK1S360ph detected at the outer edges of the islets, possibly in alpha cells (arrows, Fig 3E).

### Pharmacological inhibition of the Chromatin modifier MSK1/2 using SB-747651A

Next, we pharmacologically inhibited the MSK1/2 using SB-747651A, the best-available specific and potent ATP-competitive inhibitor of N-terminal kinase domain of MSK1/2 [69]. Other drugs such as H89 and Ro 31–8220 have been described before for pharmacological inhibition of MSK proteins [9–20]. Naqvi et al. did a comparative study to analyze the inhibitory effect of H89, Ro 31–8220 and SB747651A, against more than one hundred kinases. They showed that SB747651A inhibits the activity of N-terminal kinase domain of MSK1 activity potently, with 5% activity remaining, as opposed to H89, which has 25% MSK1 activity remaining and Ro 31–8220 which has relatively many more off-targets. Even though we cannot discount the possibility that SB-747651A may block other related AGC kinases, based on the reagents available, this is the best method to temporally and spatially block MSK activity. SB-747651A markedly reduced the evident levels of MSK1S360ph, confirming that it effectively blocks the phosphorylation of the N-terminal domain of MSK1 (Fig 3A and 3B; n≥5 pancreata for each biological replicate, n = 2 biological replicates, [69]. An antibody against the phosphorylated N-terminal domain of MSK2 is unavailable.

Next, we performed western blot to determine whether the levels of H3S28ph and H3S10ph were altered upon SB-747651A treatment during pancreatic development. Upon SB-747651A treatment, in E15.5 pancreatic explants for one day, the levels of H3S28ph were reduced by about one third (Fig 3G–3I, n = 2 biological replicates), while H3S10ph levels remained unaltered (Fig 3H and 3J). The residual H3S28ph in SB-747651A treated explants could be from cells undergoing mitosis, which remains unchanged between controls and inhibitor treated pancreata (S6A–S6C Fig). Thus, SB-747651A impedes MSK mediated phosphorylation of histone H3S28, but not H3S10ph, in E15.5 pancreatic explants. In accord with SB-747651A inhibiting MSK mediated phosphorylation of H3 at Ser28, we examined H3S28ph and H3S10ph levels in *Msk1*; *Msk2* double mutant pancreata. We found that H3S28ph, but not H3S10ph, levels were significantly reduced in *Msk1; Msk2* double mutants (S3A–S3D Fig). This further confirms that MSK proteins mediates phosphorylation at histone H3 ser28 residue during pancreatic development. However, *Msk1-/-* mutants showed much higher induction of H3S28ph, suggesting a compensatory mechanism (S3 Fig and Fig 3A–3D). MSK1/2 has not been associated with histone modifications other than H3S28ph and H3S10ph [1].

### SB-747651A treatment augments the endocrine lineage

To determine the consequences of MSK1/2 inhibition on different pancreatic lineages, we treated E12.5 and E15.5 pancreatic explants with SB-747651A. To verify whether development of all pancreatic lineages occurs normally in the explants, we examined select genes expressed in different lineages through 4 days of explant culture. The genes specific for endocrine lineages, such as *Ins1*, *Ins2* and *Gcg*, and specific for the acinar lineage, such as *Amyl*, showed a steady increase over a period of 4 days of explant culture from E12.5 and E15.5 stages, similar to normal pancreatic development, suggesting that whole pancreatic explant cultures are compatible with differentiation of all pancreatic lineages (S4A–S4D Fig, n≥10 pancreata for each biological replicate, n = 2 biological and n = 3 technical replicates).

**Fig 4:**
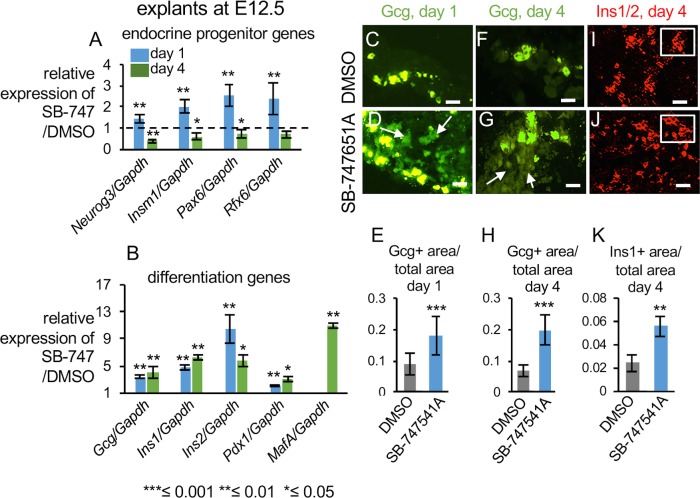
SB-747651A treatment causes increased differentiation of E12.5 pancreatic progenitors towards the endocrine lineage. (A) RT-qPCR analysis of indicated endocrine progenitor genes on day 1 (blue) and day 4 (green) of SB-747651A treatment in pancreatic explants from E12.5. Y-axis shows normalized enrichment in SB-747651A treated relative to DMSO controls. The P-values for day1 are ≤0.01 for all genes examined, for day4 are 0.008, 0.02, 0.04, 0.1 respectively for *Neurog3*, *Insm1*, *Pax6*, and *Rfx6*. n≥10 pancreata were pooled for each RT-qPCR experiment for DMSO and SB-747651A treatment and for each biological replicate, n = 2 biological and n = 3 technical replicates for each. (B) RT-qPCR analysis of indicated endocrine differentiation genes on day 1 (blue) and day 4 (green) of SB-747651A treatment in pancreatic explants from E12.5. Y-axis shows normalized enrichment in SB-747651A treated explants relative to DMSO controls. The P-values for day1 are ≤0.01 and for day4 are 0.01, 0.002, 0.05, 0.03 for *Gcg*, *Ins1*, *Ins2*, and *Pdx1* respectively. n≥10 pancreata were pooled for each RT-qPCR experiment for DMSO and SB-747651A treatment and for each biological replicate, n = 2 biological and n = 3 technical replicates for each. (C-H) Immunohistochemistry and quantification of Glucagon positive domain in pancreatic sections upon one day (C-E, P-value = 0.001) and 4 days of SB-747651A treatment (F-H, P-value ≤0.0001), n≥5 pancreata were analyzed for immunohistochemistry for each biological replicate and for each condition, DMSO and SB-747651A treatment. n = 2 biological replicates Scale bar = 50μm. The panels C and D were exposed to reveal cells expressing lower levels of Gcg. (I-K) Immunohistochemistry and quantification of Insulin1/2 positive domain in pancreatic sections upon SB-747651A treatment on day4 at E12.5 stage, P-value, 0.005, n≥5 pancreata for each biological replicate and for each condition, DMSO and SB-747651A treatment. n = 2 biological replicates, scale bar = 25μm. Insets on panels I and J shows magnified views to highlight cytoplasmic staining of Ins1/2. All area measurements were done relative to the total pancreatic area per section. Arrows in D, G show low level expression of Glucagon in explants treated with SB-747651A treatment. Values are averages of 3 independent experiments ± standard error unless otherwise stated. All P-values were calculated by Student’s t test, comparing DMSO and SB-747651A treated samples in independent experiments, *≤ 0.05, **≤ 0.01, ***≤0.001, two-tailed.

SB-747651A treatment for one day in E12.5 explants caused an increase in the transcript levels of the endocrine progenitor marker *Neurog3* by about 1.5 fold and its immediate downstream targets *Insm1*, *Pax6*, and *Rfx6* by 2-fold (Fig 4A, P-values ≤0.01, n≥10 pancreata each for DMSO and SB-747651A treatment for each biological replicate, n = 2 biological and n = 3 technical replicates for each). Continued SB-747651A treatment for a total of 4 days resulted in downregulation of *Neurog3*, *Insm1*, *Pax6*, and *Rfx6* mRNAs (Fig 4A, P-value are 0.008, 0.02, 0.04, 0.1 respectively). The genes specific for the different endocrine lineages *Gcg*, *Ins1*, *Ins2*, and *Pdx1* were increased 3-fold, 4-fold, 10-fold, and 2-fold respectively on day 1 (Fig 4B, n≥10 pancreata each for DMSO and SB-747651A treatment and for each biological replicate, n = 2 biological and n = 3 technical replicates, all P-values ≤0.01, respectively). In contrast to endocrine progenitor markers, the levels of endocrine differentiation genes *Gcg*, *Ins1*, *Ins2*, and *Pdx1* remained upregulated on day 4 of continuous inhibitor treatment (Fig 4B, n≥10 pancreata each for DMSO and SB-747651A treatment and for each biological replicate, P-value are 0.01, 0.002, 0.05, 0.03, respectively). This suggests that at the E12.5 stage, there are limited number of competent pancreatic progenitors that can differentiate towards Neurog3 positive endocrine lineages. These limited number of Neurog3 positive pancreatic progenitors are exhausted and precociously differentiate towards specific endocrine lineages upon SB-747651A treatment. *MafA*, a mature beta cell differentiation marker, showed about 10-fold enrichment upon continuous SB-747651A treatment for 4 days (Fig 4B, n≥10 pancreata each for DMSO and SB-747651A treatment and, n = 2 biological and n = 3 technical replicates for each biological replicate P-value = 0.01). Together, these data indicate that prolonged treatment with SB-747651A enhances endocrine differentiation in E12.5 explants (Fig 4B).

Next, immunohistochemistry was performed to determine if the above-mentioned changes in transcript levels in the E12.5 explants were due to increased numbers of endocrine cells. A more than 2-fold increase in the overall glucagon expression domain was observed at day 1 of culture (Fig 4C–4E, P-value = 0.001, n≥5 pancreata for each biological replicate and for each condition, DMSO and SB-747651A treatment, n = 2 biological replicates) and also on day 4 of culture (Fig 4F–4H, P-value ≤0.0001, n≥5 pancreata for each biological replicate and for each condition, DMSO and SB-747651A treatment, n = 2 biological replicates). Additionally, cells expressing lower levels of Glucagon (indicated by arrows, Fig 4D and 4G), in addition to normal bright Glucagon cells, were observed upon SB-747651A treatment. These cells could not be visualized in DMSO controls (compare Fig 4 panels C and D and Fig 4 panels F and G). The beta-cell expression domain indicated by Insulin1/2 increased by 2.3 fold upon SB-747651A treatment on day 4, with individual cells appearing equally bright in SB-747651A treatment versus DMSO controls (Fig 4I–4K, P-value, 0.005, n≥5 pancreata for each biological replicate and for each condition, DMSO and SB-747651A treatment, n = 2 biological replicates). We conclude that SB-747651A treatment causes increased differentiation of E12.5 progenitors towards the endocrine lineage.

**Fig 5:**
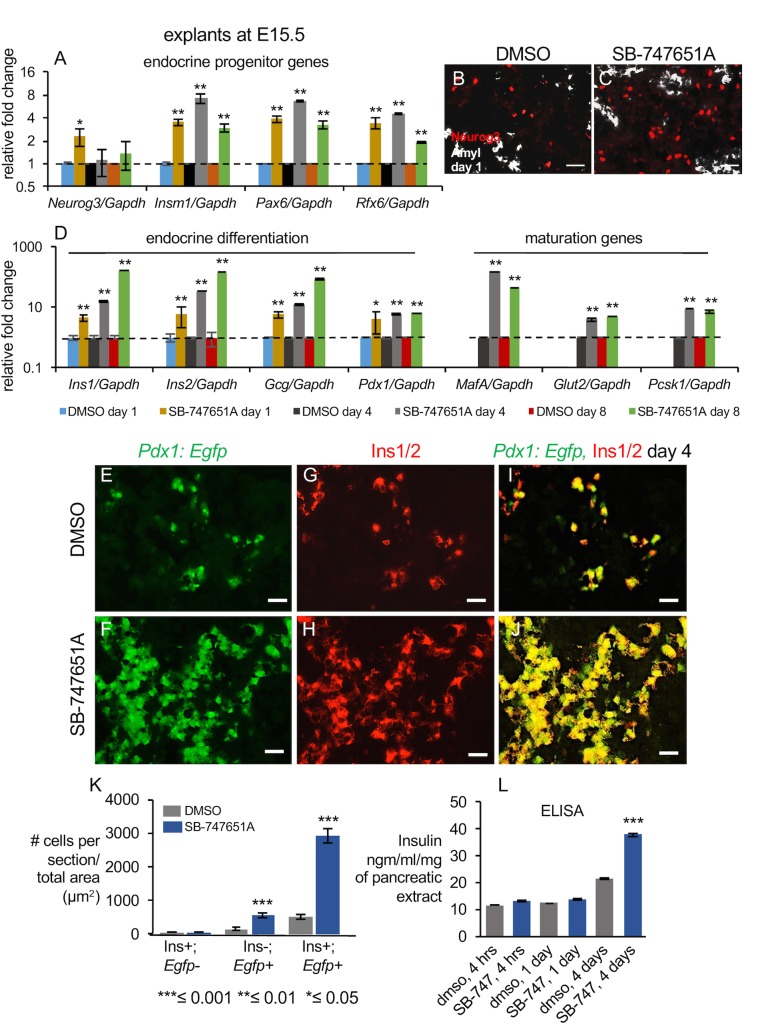
Pancreatic beta cell fate is markedly increased by SB-747651A treatment at E15.5. (A) Expression levels of indicated endocrine progenitor genes on days 1, 4, and 8 of SB-747651A treatment in explants harvested from E15.5 stage. Y-axis shows relative fold change in DMSO controls and SB-747651A treated explants. n≥10 pancreata were pooled for both DMSO and SB-747651A treated explants for each biological replicate, n = 2 biological replicates. P-values for Neurog3, day 1 = 0.03 and for *Insm1*, *Pax6*, and *Rfx6* were ≤0.01 for both days 4 and 8. (B, C) Immunohistochemistry on pancreatic sections from E15.5 explants cultured for one day in DMSO or SB-747651A showing expression of Amyl and Neurog3. n≥5 pancreata for each biological replicate and for each condition, DMSO and SB-747651A, n = 2 biological replicates, P-value = 0.008. Amylase is pseudo-colored white to enhance contrast. (D) Expression levels of indicated endocrine differentiation and indicated mature genes in E15.5 stage explants cultured for 1, 4, and 8 days. Y-axis shows relative fold change in DMSO and SB-747651A treated explants. The color scheme for the bar graphs is displayed in the figure. n≥10 pancreata were pooled each for DMSO and SB-747651A treatment for each biological replicate, n = 2 biological replicates. P-values for *Ins1*, *Ins2*, and *Gcg* on day1 are ≤0.001, ≤0.01 for day 4 and ≤0.01 for day 8, P-value ≤0.01 for *MafA*, *Glut2* and *Pcsk1* on days 4 and 8. (E-K) Expression and quantification of Insulin1/2 visualized by immunohistochemistry on pancreatic sections from *Pdx1*:*Egfp* pancreata, obtained from E15.5 stage, cultured for 4 days in DMSO or SB-747651A. P-values = 3.12×10^−7^ for Pdx1:Egfp+; Ins1+ and 5.6×10^−5^ for *Pdx1*:*Egfp+*; Ins1- on panel H, n≥5 pancreata each for DMSO and SB-747651A treatment and for each biological replicate, n = 3 biological replicates. Scale bar on all panels = 25μm. Values are averages of independent experiments ± standard error unless otherwise stated. (L) Insulin quantification by ELISA in pancreatic lysates treated with DMSO or SB-747541A for 1 or 4 days. All P-values were calculated by Student’s t test, comparing DMSO and SB-747651A treated samples in 3 independent experiments, *≤ 0.05, **≤ 0.01, ***≤0.001, two-tailed. All RT-qPCR values are normalized to beta-actin.

SB-747651A treatment on explants harvested at the E15.5 stage led to more robust increases in endocrine lineage and beta cell specific transcripts. *Neurog3* transcripts were enriched 1.7 fold on day 1 (Fig 5A, n≥10 pancreata each for DMSO and SB-747651A treatment and for each biological replicate, n = 2 biological replicates, P-values = 0.03 for day 1). Immunohistochemistry revealed 2-fold more Neurog3 positive cells upon SB-747651A treatment, as compared to controls on day 1 (n≥5 pancreata for each biological replicate and for each condition, DMSO and SB-747651A treatment, n = 2 biological replicates, P-value = 0.008, arrowheads, Fig 5B and 5C and S5A–S5F Fig). The *Neurog3* transcripts returned to wild type levels on days 4 and 8 (Fig 5A). More strikingly, the immediate downstream targets of *Neurog3*, such as *Insm1*, *Pax6*, and *Rfx6*, were enriched on days 1, 4, and 8 by 4–8 fold (Fig 5A, n≥10 pancreata each for DMSO and SB-747651A treatment and for each biological replicate, n = 2 biological replicates, P-values ≤0.01).

The genes expressed in specific endocrine lineages such as *Ins1*, *Ins2*, and *Gcg* transcripts increased by 3–5 fold on day 1 (Fig 5D, n≥10 pancreata for each biological replicate for DMSO and SB-747651A treatment, n = 2 biological replicates, all P-values ≤0.001), rising to 16–33 fold on day 4 (Fig 5D, n≥10 pancreata for each biological replicate for DMSO and SB-747651A treatment, n = 2 biological replicates, all P-values ≤0.01) and 86–163 fold on day 8 of SB-747651A treatment in E15.5 explants (Fig 5D, n≥10 pancreata for each biological replicate for DMSO and SB-747651A treatment, n = 2 biological replicates, all P-values ≤0.01). The *Ins1*, *Ins2*, and *Gcg* transcripts were 2.5, 5.2, and 2.3 fold more induced, respectively, upon SB-747651A treatment at E15.5 than at E12.5 (S4E–S4J Fig), consistent with the later-born (E15.5) pancreatic progenitors gaining competence to generate more beta cells, agreeing with previous studies [51]. Mature beta cell genes including *MafA* increased by 120 fold on day 4 (P-value = 0.01), while *Glucose Transporter2* (*Glut2*) and *Proconvertase1* (*Pcsk1*), genes associated with glucose transport and insulin processing increased by about 5-fold and by ~8 fold in SB-747651A treated explants at days 4 and 8 of explant culture from E15.5 (Fig 5D, n≥10 pancreata each for DMSO and SB-747651A treatment and for each biological replicate, n = 2 biological replicates, P-values less than 0.01 for *Glut2* and *Pcsk1*). Altogether, these data indicate that SB-747651A treatment dramatically augments the expression of beta cell specific genes at E15.5.

Consistent with changes in transcript patterns, immunofluorescence showed a remarkable 5-fold increase in the expression domains of double positive Ins1/2+; Pdx1:Egfp+ cells (Fig 5E–5K, P-value = 3.12×10^−7^, n≥5 pancreata for each biological replicate for DMSO and SB-747651A treatment, n = 3 biological replicates) and more than a 3-fold increase in *Pdx1*:*Egfp+*; Ins1/2- cells, in SB-747651A treated E15.5 pancreatic explants on day 4 (P-value = 5.6×10^−5^, Fig 5E–5K, n = 3 biological replicates). Extensive co-localization was observed between Pdx1 protein levels and Insulin1/2 at E15.5 stage of pancreatic development (S2G and S2H Fig. Next, we determined the overall levels of processed form of Insulin by an ELISA assay. The ELISA assay revealed more than 2-fold elevation in processed Insulin levels in lysates from pancreas treated with SB-747541A for 4 days (Fig 5L, P-value = 0.003). Altogether, these data indicate SB747541A treatment causes marked increase in the production of Insulin in pancreatic explants.

**Fig 6:**
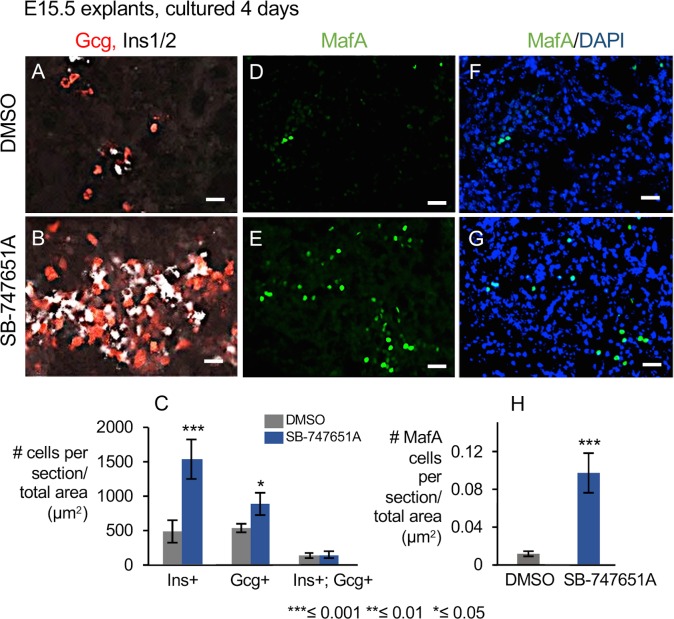
Expression analysis of Ins1/2; Gcg and MafA upon SB-747541A treatment. (A-C) Co-expression and quantification of Ins1/2 and Gcg expression domains by immunohistochemistry on pancreatic sections from E15.5 stage explants cultured for 4 days in DMSO or SB747541A. P-values = 4×10^−4^ for Ins1/2+;Gcg- and 0.02 for Gcg+;Ins1/2-, n≥5 pancreata each for DMSO and SB-747651A treatment and for each biological replicate, n = 3 biological replicates. (D-H) Expression and quantification of MafA positive cells in pancreatic explants treated with DMSO (D, F) and SB-747541A (E, G) for 4 days. Scale bar on all panels = 25μm. Values are averages of 2 independent experiments ± standard error unless otherwise stated. All P-values were calculated by Student’s t test, comparing DMSO and SB-747651A treated samples in 2 independent experiments, *≤ 0.05, **≤ 0.01, ***≤0.001, two-tailed.

Next, we performed co-immunohistochemical staining for both Insulin and Glucagon by a method that ensures minimal cross-reactivity between antibodies (See Materials and Methods). This is important because Ins1/2;Gcg double positive cells arise during early pancreatic development and do not contribute to mature islets [50]. Ins1/2+;Gcg- were induced 4-fold (Fig 6A–6C, P-value = 4×10^−4^ and S2 M-T, n = 3 biological replicates) and Gcg+;Ins1/2- were induced about 2.2 fold (Fig 6A–6C, P-value = 0.02) upon SB-747651A treatment, while Ins1/2+;Gcg+ double positive cells remained the same as the controls (Fig 6A–6C, P-value = 0.3). Altogether, our data indicate that inhibition of MSK1/2 using SB-747651A drug markedly promotes the differentiation of alpha and beta cells independently without any co-induction of Ins1/2 and glucagon. This suggests that SB-747651A treatment induces mature alpha and beta cells that contribute to adult islets, as suggested by lineage tracing studies of Herrera 2000 [50], instead of double positive Ins+;Gcg+ cells that do not contribute to adult islets.

Next, we examined MafA, a mature beta-cell marker by immunofluoroscence to confirm our previous observation that continuous SB-747541A treatment for 4 days in E15.5 explants causes nearly 100-fold increase in the MafA transcripts (Fig 5D). Immunofluorescence revealed nearly 10-fold increase in the number of MafA cells upon SB-747541A treatment compared to DMSO controls (Fig 6D–6H, P-value = 0.003, n = 4 independent pancreata) confirming that the number of cells expressing MafA were elevated in these conditions.

### The acinar lineage is diminished upon SB-747651A treatment

**Fig 7:**
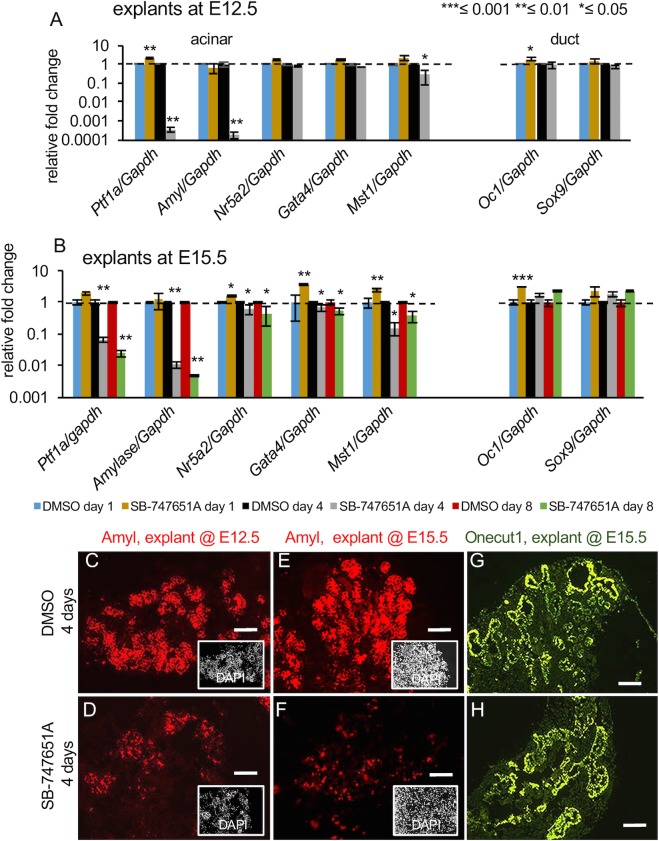
Acinar fates are markedly depleted upon SB-747651A treatment. (A) Expression levels of indicated acinar and duct genes upon culture of E12.5 explants for 1 and 4 days. Y-axis shows relative fold change in DMSO controls and upon SB-747651A treatment. P-values ≤0.01 for both *Ptf1a* and *Amyl* for day 4, n = 3 biological and 3 technical replicates. (B) Expression levels of indicated acinar and duct genes upon culture of E15.5 explants for 1 day, 4 days, and 8 days on explants treated with DMSO or SB-747651A (n = 3 independent replicates). Y-axis shows relative fold change in DMSO controls and upon SB-747651A treatment. Color scheme for the bar graphs is displayed in the figure. n≥10 pancreata for each each biological replicate for DMSO and SB-747651A treatment, P-values ≤0.01 for *Ptf1a* and *Amyl* for day 4 and day8, n = 3 biological and 3 technical replicates. (C-F) Immunohistochemistry showing Amyl expression on pancreatic sections from E12.5 (C, D, scale bar = 50μm) and E15.5 (E, F, scale bar = 50μm) stages harvested at 4 days of culture. n≥5 pancreata for each biological replicate for DMSO and SB-747651A treatment n = 2 biological replicates. (G, H) Oc1 expression in sections from pancreatic explants from E15.5 stage (scale bar = 100μm, G, H) from DMSO or SB-747651A treated explants, harvested at 4 days of culture. Insets shows pseudo-colored DAPI staining. Values are averages of independent experiments ± standard error unless otherwise stated. All P-values were calculated by Student’s t test, comparing DMSO and SB-747651A treated samples in independent experiments, *≤0.05, **≤0.01, ***≤0.001, two-tailed.

In contrast to the endocrine lineage, the acinar lineage was severely depleted upon continued SB-747651A treatment. After a slight induction on day 1, exocrine genes such as *Ptf1a*, *Amyl*, *Nr5a*2, *Gata4*, and *Mst1* were drastically reduced by 3125, 5263, 1.2, 1.4, and 3.3 fold, respectively, upon SB-747651A treatment for 4 days in E12.5 explants (Fig 7A, n≥10 pancreata each for DMSO and SB-747651A treatment and for each biological replicate, P-values ≤0.01 for *Ptf1a* and *Amyl*, n = 3 biological and 3 technical replicates). SB-747651A treatment in E15.5 explants led to corresponding 15, 100, 1.7, 1.5, and 6.7 fold reductions at day 4 and 43, 204, 2.3, 1.9, and 2.7 fold reductions, respectively, at day 8 of culture for *Ptf1a*, *Amyl*, *Nr5a*2, *Gata4*, and *Mst1* respectively (Fig 7B, n≥10 pancreata for each biological replicate for DMSO and SB-747651A treatment, P-values ≤0.01 for *Ptf1a* and *Amyl*, unless otherwise indicated on the graphs, n = 3 biological and 3 technical replicates). Note that the maximum fold decrease was found for *Ptf1a* and *Amyl*, but not for *Nr5a1*, *Gata4* and *Mst1*, suggesting that response elements in the *Ptf1a* and *Amyl* genes are most sensitive to SB-747651A treatment.

Consistent with the changes in the transcript levels, Amylase protein levels were substantially reduced in explants from E12.5 and E15.5 stages that were cultured for 4 days (Fig 7C–7F, n≥5 pancreata for each biological replicate for DMSO and SB-747651A treatment and, n = 2 biological replicates). The E12.5 stage is associated with the initiation of acinar differentiation, while significant acinar differentiation has occurred by the E15.5 stage, suggesting that MSK1/2 contributes to both specification and maintenance of acinar fates.

To determine whether newly recruited endocrine cells are induced within the peripheral acinar domain at E15.5 upon SB-747651A treatment, we examined the expression of Neurog3 and Amyl on day 1 in SB-747651A treatment explants. We considered this because the *Amyl* positive acinar domain is evident on day 1 of SB-747651A treatment as opposed to day 4 when acinar domain is essentially lost (Fig 7A and 7B, day 1). Any co-induction of Neurog3 with Amyl would potentially indicate trans-differentiation of acinar cells into endocrine lineage. However, we did not detect any co-expression between Amyl and Neurog3 on day 1 of SB-747651A treatment (Fig 5B and 5C and S5A–S5F Fig) suggesting that SB-747651A -induced endocrine cells may not be arising within the peripheral acinar domain at E15.5. More detailed lineage tracing studies would further resolve this.

The trunk and future duct markers *Oc1* and *Sox9* were slightly induced upon SB-747651A treatment at E12.5 and E15.5, but returned to wild type levels upon continuous culture in SB-747651A (Fig 7A and 7B). Immunohistochemistry also did not reveal changes in the Oc1 expression domain upon SB-747651A treatment in E15.5 explants (Fig 7G and 7H). In conclusion, SB-747651A treatment significantly depletes the acinar lineage without affecting duct fates.

### SB-747651A treatment does not lead to significant changes in cell proliferation or apoptosis

To investigate whether increased proliferation contributes to enhanced specification of endocrine cells upon SB-747651A treatment, we administered BrdU on E15.5 explants for 8 hrs on day 1 and examined the explants after 2 days. Quantification of the number of BrdU positive nuclei, normalized to total explant surface area, showed no change between DMSO controls and SB-747651A treated cells indicating that no net gain in proliferation contributes to increased endocrine expression domain (S6A–S6C Fig, P-value = 0.37, n≥7 pancreata each for DMSO and SB-747651A treatment and for each biological replicate, n = 2 biological replicates)

Next, we examined apoptosis in SB-747651A treated explants using two independent methods: Cleaved Caspase3 and Tunel staining. We did not find significant changes in the number or location of cleaved caspase3 or TUNEL positive cells in SB-747651A treated explants, on either day 1 (S6D–S6I Fig) or day 4 (S6J–S6O Fig), n≥7 pancreata each for DMSO and SB-747651A treatment and for each biological replicate, n = 2 biological replicates) of explant cultures, indicating that depletion of acinar fates observed upon SB-747651A treatment may not be attributable to apoptosis.

### Repression of endocrine fates by MSK1/2 is independent of intercellular interactions in the explants

**Fig 8:**
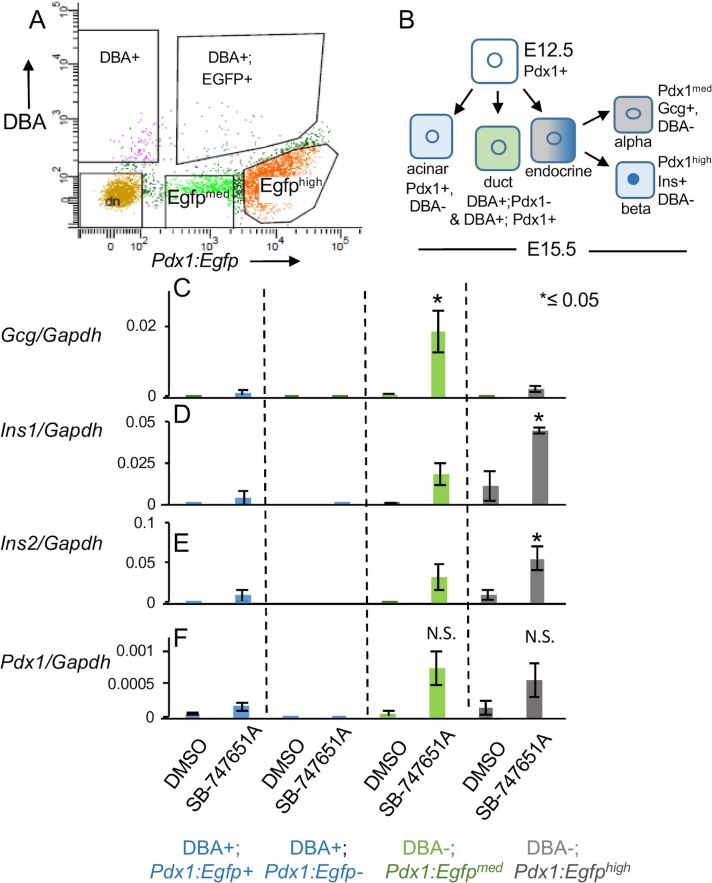
Enhancement of endocrine genes upon SB-747541A treatment is independent of intercellular interactions. (A) Scatter plos of cells from E15.5 pancreata, sorted by FACS into Egfp and rhodamine conjugated DBA positive cells. (B) Hierarchy of different lineages during pancreatic development indicating expression of DBA and Pdx1-Egfp. (C-F) Normalized expression of indicated genes on third day of culture of different FACS sorted populations treated with either DMSO or SB-747651A. n≥25 pooled pancreata for each biological replicate, n = 2 biological and 3 technical replicates, P-values = 0.01 for *Gcg* in DBA-;*Pdx1*:*Egfp*^*med*^, 0.01 and 0.009, respectively for *Ins1* and *Ins2*, in DBA-;*Pdx1*:*Egfp*^*high*^ cell sub-population. Values are averages of independent experiments ±standard error unless otherwise stated. All P-values were calculated by Student’s t test, comparing DMSO and SB-747651A treated samples in independent experiments, *≤0.05, **≤0.01, ***≤0.001, two-tailed.

To determine whether the observed enhancement of endocrine differentiation upon SB-747651A treatment is an indirect consequence of reduced acinar differentiation in the explants due to intercellular interactions, we set up dilute cultures of completely dissociated and FACS-isolated pancreatic epithelial cells, plated at a sub-clonal density of 2000–3000 cells/ 2 cm^2,^. Representative images illustrating well-separated single cells on day 1 of culture are shown in S2–S2L Fig. We sorted for DBA, a cell surface antigen expressed on the duct cells [70, 71] and *Egfp* in *Pdx1*:*Egfp* positive pancreata (Fig 8A and S7A Fig). We found that the enrichment profiles of genes examined in this study for the different cell subpopulations remained similar upon 3 days of culture on OP9 feeder cells ([71], S7B and S7C Fig).

The following four populations were parsed out using FACS (Fig 8A and 8B and S7A Fig, n≥25 pooled pancreata for each biological replicate, n = 2 biological and 3 technical replicates): DBA+; *Pdx1*:*Egfp*- duct progenitors [71], DBA+;*Pdx1*:*Egfp*+, a mix of duct and endocrine progenitors (S7B and S7C Fig, *Oc1+*, *Neurog3+*, *Pdx1+*), DBA-;*Pdx1*:*Egfp*^*med*^, a mix of duct, alpha cell, and endocrine progenitors (S7B and S7C Fig, *Oc1+*, *Gcg+*, *Neurog3+*) and DBA-;*Pdx1*:*Egfp*^*high*^, a mix of beta and acinar cell precursors (S7B and S7C Fig, *Ins1+*, *Ins2+*, *Amyl+*). It is not clear why DBA+; *Pdx1*:*Egfp*- cells aren’t as enriched for Oc1 as the double positive DBA+; Pdx1:Egfp+ population. Prior studies have shown that Oc1 is co-expressed with a subset of Neurog3 positive cells in the trunk domain at E15.5, explaining its enrichment in the DBA+;*Pdx1*:*Egfp*+ and DBA-;*Pdx1*:*Egfp*^*med*^ populations (S7B and S7C Fig, 42].

The alpha cell specific *Gcg* transcripts were maximally upregulated upon SB-747651A treatment in DBA-;*Pdx1*:*Egfp*^*med*^ cells (Fig 8C, P-value = 0.01) while the beta cell specific *Ins1* and *Ins2* transcripts were induced maximally from DBA-; *Pdx1*: *Egfp*^*high*^ cells upon SB-747651A treatment (Fig 8D and 8E, P-value = 0.01, 0.009 respectively, n≥25 pooled pancreata for each biological replicate, n = 2 biological and 3 technical replicates). The DBA+;*Pdx1*:*Egfp*+ double positive population and DBA+; *Pdx1*:*Egfp*- cells showed a modest induction of endocrine hormones. Altogether, these data indicate that activation of endocrine genes upon SB-747651A treatment is independent of intercellular interactions.

### MSK1 and MSK2 proteins have distinct roles during pancreatic development

**Fig 9:**
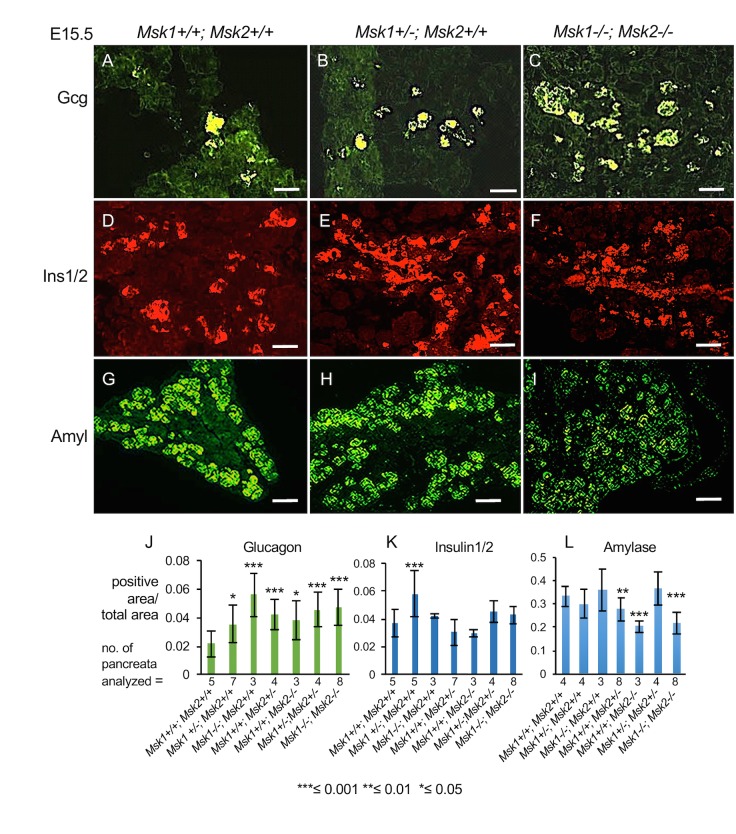
MSK1 and MSK2 proteins have distinct functions during pancreatic development. (A-I) Representative images showing expression of Glucagon (A-C, scale bar = 50μm), Insulin (D-F, scale bar = 50μm) and Amylase (G-I, scale bar = 100μm,) in the indicated genotypes at E15.5. (J-L) Glucagon, Insulin, and Amylase positive areas normalized to total area in the indicated genotypes at E15.5. For Gcg, P-values are 0.01 for *Msk1+/-*, 3.3x10^-7^ for *Msk1-/-*, 2.2x10^-4^ for *Msk2+/-*, 0.02 for *Msk2-/-*, 2.2x10^-6^ for *Msk1+/-*; *Msk2+/-* and 3.1x10^-5^ for *Msk1-/-;Msk2-/-*. For Ins1, P-value = 1.5x10^-5^ for *Msk1+/-*. For Amyl, P-values are 3.3x10^-13^ for *Msk2+/-*, 0.004 for *Msk1+/-*; *Msk2+/-*, 1.7 x10^-6^ for *Msk1-/-*;*Msk2-/-*. Average number of sections/pancreas for Ins1 and Gcg respectively were 17, 9.8 (wild type), 17.83, 5.6 (*Msk1+/-*), 15.6, 5 (*Msk1-/-*), 16.3, 5 (*Msk2-/-*), 14.75, 6.375 (*Msk2+/-*), 13, 13.5 (*Msk1+/-; Msk2+/-*), 15.12, 6.75 (*Msk1-/-; Msk2-/-*). All P-values were calculated by Student’s t test, relative to wild type controls, *≤0.05, **≤0.01, ***≤0.001, two-tailed unequal variance, two-tailed. Values are averages of independent experiments ± standard error unless otherwise stated.

To assess whether genetic ablation of MSK1/2 may agree with pharmacological inhibition, we analyzed pancreata of germline *Msk1* and *Msk2* knockouts at E15.5 [9–12]. According to our previous observations (Figs 4, 5, 6 and 7), inhibition by SB-747541A at E15.5 stage yielded most robust increases in endocrine cell specification. Hence, this stage was chosen for further analysis of *Msk1/2* mutants. Compared to the wild type, an increase in total alpha cell mass was observed in all of the genotypes examined (Fig 9A–9C and 9J and S8A–S8E and S8U Fig). The increases in alpha cell mass were 1.6 fold for *Msk1+/-* (Fig 9A, 9B and 9J and S8A, S8B and S8U Fig, P-value = 0.02), 2.6 fold for *Msk1-/-* (S8C and S8U Fig, P-value = 3.3x10^-7^), 1.9 fold for *Msk2+/-* (S8D and S8U Fig, P-value = 2.2x10^-4^), 1.7 fold for *Msk2-/-* (Figs 9J and S9D, P-value = 0.02), 2.1 fold for *Msk1+/-*; *Msk2+/-* (S8U Fig, P-value = 2.2x10^-6^ and S9D Fig), and 2.5 fold for *Msk1-/-;Msk2-/-* (Fig 9C and 9J and S8E and S8U Fig, P-value = 3.1x10^-5^). Notably, the fold increase in alpha cell mass in Msk knockouts are similar to the ones obtained upon SB-747651A treatment in E12.5 explants (Fig 4C–4H). We did not observe any overall changes in the pancreatic size at this stage of development.

In *Msk1+/-* pancreata, a 1.6 fold increase in beta cell mass was observed (Fig 9D, 9E and 9K and S8F, S8G and S8V Fig, P-value = 1.5x10^-5^), while other genotypes had only a nominal increases in beta cell mass (Fig 9K and S8H–S8J and S8V Fig). These observations indicate that knockout of both *Msk1* and *Msk2* promotes specification of alpha cell fates while only monoallelic loss of *Msk1* promotes beta cell mass. A conditional knockout of *Msk* isoforms in the pancreas at E15.5 stage would be useful in resolving the differences observed in beta-cell induction in SB-474541A treated pancreatic explants at E15.5 (about 6-fold) versus global *Msk* knockouts (Compare Figs 5K and 9K).

Conversely, for acinar differentiation, *Msk2* heterozygous, *Msk2* homozygotes, and *Msk1-/-*;*Msk2-/-* double mutant pancreata showed significantly reduced Amylase positive expression domains (Fig 9G–9I and 9L, S8K, S8N, S8O, S8W and S9F Figs, P-value = 3.3x10^-13^, 0.004, 1.7 x10^-6^ for *Msk2+/*-, *Msk2* homozygotes, *Msk1-/-*;*Msk2-/-* double mutant pancreata) while *Msk1* heterozygous or homozygous mutants did not show significant changes in the Amylase domain (S8K–S8M and S8W Fig). In addition, the intensity of the Amylase expression showed a considerable reduction in the *Msk1; Msk2* double mutant pancreata. These results suggest that both *Msk1* and *Msk2* function redundantly in stimulating acinar specification, with the *Msk2* isoform playing a more dominant role during acinar differentiation.

## Discussion

In this study, we uncovered a novel function for the chromatin modifier, MSK1/2, in suppressing endocrine and promoting acinar differentiation during pancreatic development. We initiated this study by performing a screen for signaling-dependent histone modifications at sentinel pancreatic genes in FACS-enriched cell subpopulations. The screen led us to examine the dynamics of H3S28ph in progenitors isolated at different developmental stages. We identified an enrichment of H3S28ph, which can be induced by MAPK, RA, PI3K, TNF, Jak-Stat, or cAMP/CREB signaling pathways in different cellular contexts (Table 1), at endocrine and acinar lineage-specific genes in the multipotent pancreatic progenitors, before the cells’ overt differentiation into respective lineages. Both the *Pdx1* promoter and *Pdx1*^*enh*^, and acinar specific *Ptf1a* and *Amyl* gene promoters are pre-marked with H3S28ph in E12.5 progenitors. These data indicate that before the overt differentiation of multipotent progenitors to different pancreatic lineages, the endocrine and the acinar genes are pre-marked with H3S28ph in the progenitors. This may relate to the developmental competence of the progenitors as it might set up these loci for further activation or repression [2, 7] depending on whether H3S28ph associated proteins are transcriptional activators or repressors at a given loci [19, 20]. To further resolve this, we inhibited the corresponding chromatin modifier of H3S28ph during pancreatic development, MSK1/2.

Inhibition of the chromatin modifier for H3S28ph, MSK1/2, using SB-747541A, caused more frequent differentiation towards the endocrine fate, while acinar differentiation is suppressed. Inhibition of MSK1/2 led to the induction of *Insulin1* and *Insulin2* transcripts by 160- and 140-fold, respectively, and *Glucagon* transcripts by 85-fold on day 8 in E15.5 explants. A striking 5-fold increase was observed in the expression domain of *Ins1* positive cells in E15.5 SB-747651A treated explants. In contrast, the acinar transcripts *Ptf1a* and *Amyl* and the *Amyl* positive expression domain are severely depleted upon SB-747651A treatment at both E12.5 and E15.5. These data indicate that the normal function of MSK1/2 proteins would be to establish transcriptional activation of acinar genes such as *Ptf1a* and *Amylase2a* while repressing endocrine progenitor genes such as *Neurog3*, *Insm1*, *Pax6*, and *Rfx6*.

Considering a near ubiquitous expression of both MSK1ph and MSK2ph in the pancreatic epithelia, these data would suggest that MSK proteins normally induce acinar differentiation of the pancreatic progenitors unless they get inhibited, perhaps by other signaling pathways. The pathways that inhibit MSK activity to allow sparse induction of endocrine genes would require future experimentation. Alternatively, MSK proteins could act as repressors of endocrine genes, along with other transcriptional regulators, to fine-tune expression in the progenitors. Future studies would explore the mechanisms that underlie these effects. We also carefully examined whether Ins1/2 and Gcg where co-induced upon SB-747541A treatment to determine if we might be expanding an embryonic pool of double positive Ins1/2-Gcg cells. We did not observe a significant expansion of the double positive Ins1/2-Gcg cells. Germline knockout of MSK1 and MSK2 expanded alpha cell mass, while the *Amyl* positive acinar domain was reduced. The observation of enhanced alpha cell production in the germline MSK1/2 knockout is consistent with alpha cells being the preferential endocrine fate during the initial stages of endocrine differentiation [51]. A conditional knockout of *Msk1/2* in the pancreas at the onset of secondary transition would be most critical to define its role specifically during beta cell differentiation. Altogether, the data indicate that inhibition or knockout of MSK proteins causes more frequent differentiation to *Neurog3* positive endocrine progenitors, leading to an increase in the domain of endocrine cell types while differentiation towards the acinar lineage is suppressed.

A direct role of MSK1S360ph and MSK2T586ph proteins in modulating the endocrine and acinar cell fate choices was implied by MSK1S360ph and MSK2T586ph binding to the H3S28ph enriched promoters for *Amyl* and *Ptf1a*, and the *Pdx1*^*enh*^ in E12.5 multipotent progenitors. The presence of both H3S28ph and MSK proteins on both endocrine and acinar genes and the contrasting effects of SB-747651A treatment on acinar and endocrine genes indicate that MSK mediated H3S28ph may not be simply correlated with the transcriptional status of the genes during pancreatic development. This is similar to other studies where MSK mediated H3S28ph modification directs both positive and negative influences on the gene expression (See Introduction for References).

We suggest that SB-747651A treatment of pancreatic progenitors arising from embryonic stem cells could be used to enhance the production of beta cells for type I diabetic patients. Our approach, to assess chromatin state dynamics at different developmental stages, proved useful in identifying a drug-targetable step to enhance beta cell development. Similar approaches, e.g. starting with the substrates in Table 1, could be used in other developmental and stem cell differentiation contexts to enhance the production of desired cell fates.

## Materials and Methods

### Immunostaining of single cell suspensions for FACS sorting

Pancreata from 20–25 mice were dissected from E15.5 *Pdx1*:*Egfp* embryos at a given time and treated with 0.25% trypsin for 7–10 min to generate single cell suspensions, washed 3X in 10% serum + PBS, and fixed in Media A, with 2–3% formaldehyde (Invitrogen Cell and Perm kit) for 10 minutes at RT (100 μl for 10^6^ cells). Formaldehyde was quenched with 1 M glycine for 5 min followed by 3X washes with PBS. Non-specific binding of antibodies was blocked by 10% FBS + PBS for 1 hr. Cells were subsequently resuspended in permeabilization media B (100 μl for 10^6^ cells) and incubated with primary antibody (2 μg/ml) for Oc1 for 1 hr at room temperature. One tenth of the cell suspension was set aside for incubation with same concentration of rabbit IgG (2 μg/ml). Cells incubated with Oc1 and IgG antibody were washed 3x with PBS+10% FBS for 10 min at RT. Both Oc1 and IgG treated cells were incubated with fluorophore conjugated secondary antibody (7.5 μgm/ml), lacking the Fc domain, in permeabilization media B for 1 hr. Cells were washed 3x with PBS for 10 min at RT and stored at 4°C until FACS the next day. To collect a sufficient number of cells from the E12.5 embryonic stage, 3–4 week old C3H/B6 hybrid female mice were superovulated by intraperitoneal injection of PMSG (Sigma, 5 IU/mouse), followed 2 days later by HCG injection (Sigma, 5 IU/mice). The mice were immediately bred with *Pdx1*:*Egfp* homozygous males. Pancreata were harvested from E12.5 embryos and treated with 0.05% trypsin for 5 min at 37°C, followed by three washes in 10% serum + PBS, fixed with Media A (Invitrogen Cell and Perm kit) for 10 min at RT, quenched with 1 M glycine for 5 min at RT (as before), washed 3x with PBS, and FACS sorted for *Egfp* the same day.

### FACS and RT-qPCR analysis

Cells positive for EGFP (E12.5), Oc1 (E15.5), and DBA (E15.5) were sorted from *Pdx1*:*Egfp* embryos at low pressure on a 206 Diva FACS sorting machine into 1.5 ml Eppendorf tubes containing 50 μl serum. The sorting was done relative to either *Pdx1*:*Egfp* negative cells (E12.5) or IgG (E15.5). Cells immunostained for Oc1 in a *Pdx1*:*Egfp* background was sorted into two populations: Oc and Oc+. After FACS sorting, an aliquot of 5000 cells was kept for RNA analysis and the rest for chromatin studies. DBA stained cells were sorted into five populations: DBA+, DBA+; *Pdx1*:*Egfp*+, DBA-; *Pdx1*:*Egfp* ^med^, DBA-; *Pdx1*:*Egfp* ^high^ and DBA-; *Pdx1*:*Egfp*-. The cells from each of these categories were cultured as described below.

RNA was isolated from fixed cells using Magmax FFPE total nucleic acid isolation kit (Ambion Life technologies) which involves reversing crosslinks, total nucleic acid isolation, DNAse digestion, and purification of RNA. RNA was converted into cDNA using the Biorad iScript cDNA synthesis kit. cDNA preamplified with Taqman probes (up to 7–10 probes were combined in one reaction) diluted 100-fold. The preamplified cDNA was then subjected to qPCR analysis against gene-specific Taqman probes. All qPCR values were normalized to *Gapdh*.

### Chromatin Immunoprecipitation (ChIP)-qPCR

FACS sorted cells were spun down and resuspended in 300 μl of freshly prepared cell lysis buffer (3 mM MgCl_2_, 10 mM NaCl, 10 mM Tris pH 8.0, 0.1% Igepal, 1 tablet of Roche complete mini protease inhibitors, EDTA free) for 30 min on ice. The cells were subsequently spun down at 5000 rpm for 5 minutes at 4°C. The fragmented cells were resuspended in 50–500 μl nuclear lysis buffer depending on the number of cells obtained (50 mM Tris-Cl, pH 8.0, 10 mM EDTA, 1% SDS, 1 tablet of complete mini protease inhibitors, EDTA free). The suspension was incubated on ice for 15 min, flash frozen, and stored at -80°C for future analysis. The nuclear lysates containing chromatin were sonicated in a Diagenode bioruptor. Sonication was carried out in 5 min pulses (2.5 min on, 2.5 min off) followed by 5 min rest on ice. This procedure was carried out until chromatin was sonicated to 100–300 bp. Chromatin sonication was assessed by taking an aliquot of chromatin, reversing the crosslinks, and analyzing on 3% agarose gels. For chromatin immunoprecipitation (ChIP), approximately 20,000 cells were diluted at least 5-fold in ChIP buffer (Active Motif High Sensitivity kit) and incubated with 4 μg of desired antibody or IgG on an end-to-end rotator at 4°C overnight. Subsequently, ProteinG conjugated agarose beads were added to all samples and incubated on an end-to-end rotator for 3 hrs at 4°C. The chromatin samples were then loaded on ChIP filtration columns and washed 5 times with wash buffer AM1 (Active Motif High Sensitivity kit). One fifth to one tenth of the chromatin samples were kept aside for input DNA preparation. Reverse crosslinking of input and ChIP DNA was perfomed as described: samples were incubated with 1.25 M NaCl and incubated overnight at 65°C with moderate shaking. RNA was subsequently digested with 0.15 μg/μl final concentration of RNaseA (Roche) for 1 hr at 37°C. The samples were then incubated with 0.4 μgm/μl of Proteinase K (Roche) for 2 hrs at 55°C and then 2 hrs at 80°C. DNA was purified by two phenol-chloroform extractions and one aqueous phase isolation from chloroform, followed by precipitation in 0.1 M NaCl, 0.037M glycogen and 2x volume of 100% ethanol, stored at -80°C for 48 hrs, and then spun down at 13,000 rpm for 30 min. The pellet was washed with 70% ethanol, dried, and resuspended in 40 μl of elution buffer. Following antibodies were used for ChIP assays: H3S28ph (Ab5169, GR64943-1), H3K79me2 (Millipore 04–835), H3K14acetyl (07–353), H4R3me2 (Ab5823), H3K9ac (Ab10812), H3K27ac (Ab4279), H3K18ac (Ab1191-25), H3S10ph (Millipore 07–352, Ab5176), Msk1ph (Ab81294), Msk2ph (PA5-39748). All the ChIP reactions were normalized to input and calculations were done by 2 ^(Ct input-Ct antigen)/ (Ct input-Ct IgG)^ method. All the statistical analysis was performed by paired Student’s t-test comparing the ratio of enrichment of antigen over IgG to 1. Any value more than 1 indicates enrichment over IgG while a value equal to less than 1 is of no importance as it indicates negative enrichment over background (IgG). Therefore, one-tailed t test was performed with the following thresholds: *≤ 0.05, **≤0.01, ***≤0.001.

For qPCR, chromatin samples were diluted 5 times and the following recipe was used per well: 2X Sybr green (10 μl), 0.2 μM forward+ reverse primer, 5 μl diluted ChIP DNA. The forward and reverse primers used for qPCR analysis were as follows:

*Insulin1* (-400bp): forward 5’ CATATCTTGGGTTGTTGGGTATTG 3’

                        reverse 5’ CCTTCTCCATCTCTCCTTTCA C 3’

*Insulin1* (+15bp): forward 5’ CCATCAGCAAGCAGGTATGT 3’

                        reverse 5’ CTGAAAGATAGGCAGGGTTGAG 3’

*Insulin2* (-200bp): forward 5’ GCCCTTAATGGGTCAAACAG 3’

                            reverse 5’ GCTGGTGGTTACTGGGT 3’

*Onecut1* (-220bp): forward 5’ TGTACCGGGAACTAGCAACT 3’

                   reverse 5’ CTTGCTACCTCCTGGTCTTC 3’

*Neurog3* (-80bp): forward 5’ CTGCCCTTTGTCCGGAATC 3’

                    reverse 5’ GCACCACGGGCCAATCAG 3’

*Gapdh*: forward 5’ CTACCTTAAATGAGAGCCGAGAG 3’

                reverse 5’ GCTCCTAGGGTTCGATTTCTT 3’

*Amylase2a* (-300bp*)*: forward 5’ TTGGAATGGTGCAATACAAAGA 3’

                            reverse 5’ CCAACCCGTACAAGGAGAATTA 3’

*Sox9* (-64bp*)*: forward 5’ AGCGACTTGCCAACACTGATGACT 3’

                        reverse 5’ TGGTAAAGTTGTCGCTCCCACAGA 3’

*Ptf1a* (-350bp): forward 5’ AGTCTGATGATGGCATGGGAAC 3’

                          reverse 5’ TCAGAGACCGATTGGAGACATTT3’

*Pdx1enh* (area II enhancer): forward 5’ GGAAATCCTTCCCTCAAGTTTT 3’

                        reverse         5’ GTAAATTGGCTTCCATCTCGAC 3’

*Pdx1* (-340bp): forward 5’ CTACAAAATTAGACCTCCACCC 3’

                        reverse 5’ AAAGTCTACATCTCCTCTTCCC 3’

### Culture of DBA; *Pdx1*:*Egfp* FACS sorted cells at E15.5

Two to three days prior to the experiment, OP9 feeder cells were plated to a sub-confluent density in 24-well tissue culture plate. On the day of the experiment, pancreata were dissected from E15.5 embryos, treated with 0.25% trypsin followed by three washes with PBS + 10% FBS. The pancreatic cell suspension was incubated with rhodamine conjugated to DBA (1:200) at 4°C for 40 min in PBS + 10% FBS, followed by three washes and FACS sorting. Approximately 2000–3000 cells from each fluorescence category were plated on OP9 feeder layers and an aliquot of cells was kept for RNA analysis at t = 0. In addition, OP9 cells alone, with and without inhibitors, were used to assess effects of inhibitors on feeder layers. cDNA was prepared using Biorad iscript cDNA synthesis kit and qPCR analysis performed with Taqman probes using the following recipe: 5 μl 2X Taqman master mix (Applied Biosystems), 0.5 μl 20X probe (Life technology) in 10 μl reaction. Identical amounts of RNA were used for cDNA synthesis and all values were normalized to *Gapdh*.

### Explant assays

After dissection of pancreata from E12.5 or E15.5 embryos, explants were cultured at the air-liquid interface on 0.22 μm Nucleopore etched membranes. The media (1 g/L glucose DMEM, 10% FBS, 1X Pen-Strep) was changed on the second day and harvested on days 1, 4, and 8, depending on the experiment. DMSO, at the same concentration as the inhibitor treated explants, was used as controls for inhibitor treatments.

### Immunofluorescence analysis of pancreatic explants

Pancreatic explants were fixed overnight at 4°C in 4% formaldehyde. The explants were subsequently moved to 30% sucrose overnight. The tissue was then embedded in OCT freezing medium and stored at -80°C. The embryonic explants were cut in 6 μm sections, fixed, and dried on Superfrost Plus slides (Thermo Scientific) at RT for at least 4 hours and stored at -80°C. For immunohistochemistry, slides were washed in PBS+0.1% Triton (PBT), boiled in citrate buffer (10 mM Citric Acid, pH 6) (this treatment depends on the antibody), blocked for non-specific epitopes using 10% FBS+ 1% BSA + 0.1% triton in PBS, incubated overnight in primary antibody, followed by 4X, 15 min washes in PBT, incubated with secondary antibody (HRP conjugated or Alexa fluor conjugated), followed by 4X, 15 min washes in PBT. Signal was developed either using Tyramide Signal Amplification kit (Invitrogen, 1:100 dilution of substrate in TSA amplification buffer) or DAB (Vector Labs). For double staining, the sections were citrate boiled again after first round of staining and the whole protocol was repeated. For Insulin1-Glucagon (Gcg) double staining, no citrate buffer boiling was performed and the protocol was modified to avoid cross reaction in the following way: Sections were treated FIRST with goat polyclonal anti-Gcg antibody (primary), donkey anti-goat HRP (secondary) and developed using TSA reagents as mentioned above. This was followed by incubation with guinea pig anti-Insulin1 antibody at 4°C, followed by treatment with goat biotinlyated anti-guinea pig IgG overnight at 4°C and streptavidin-HRP for 2 hrs at RT, and developed by TSA reagents using a different fluorophore. The Insulin1 positive area and total area were calculated at two different thresholds using ImageJ software. Beta cell mass was calculated by dividing insulin positive area by total pancreatic area. The pictures were acquired on NIS elements software. The following antibodies were used: Msk1ph (1:200, Ab81294), Msk2ph (1:100, PA5-39748, Thermo Scientific), Insulin (1:100, Ab7842), Amylase (1:200, A8273, Sigma), Glucagon (1:1000, SC-7779 and Ab10988), Onecut1 (1:300, H-100, SC-13050), Pdx1 (1:500, Ab2031, Beta cell body consortium), Egfp (1:1000, Ab290), Neurog3 (1:100, F25A1B3-b), Cleaved Caspase3 (1:200, Cell signaling, 9661S) and anti BrDU (1:10, Roche, 11299964001), MafA (1:100, Bethyl Labs, 00352).

### Western Blotting

E15.5 explants cultured in either SB747541A or DMSO for 1 day were homogenized in hypotonic buffer (10 mM Tris, 10 mM NaCl, 3 mM MgCl_2_, 0.5% NP-40, 5 mM EDTA, 2.5 mM Na-fluoride, 2 mM sodium orthovanadate, fresh protease inhibitors from Roche) and incubated on ice for 30 min. Cell lysates were spun down at 4°C and the nuclear pellets were resuspended in RIPA buffer (0.05 M Tris, 0.15 M NaCl, 1% NP-40, 0.5% deoxycholate, 1% SDS). The nuclei were sonicated for 1 min with a Diagenode bioruptor (30 sec on, 30 sec off) and protein concentration measured by Bicinchoninic Acid assay (Pierce Biotechnology). Samples were separated on 4–12% Bis Tris polyacrylamide gels and transferred to activated PVDF membranes. The membranes were blocked with 10% dry milk in TBST (0.1% Triton) overnight, followed by primary antibody for 2 hrs or overnight, 3X 5’ washes in TBST, HRP-conjugated secondary (1:4000), 3X 5’ washes in TBST (0.1% Triton) washes and developed by Super Signal West-pico or West-femto chemiluminescent reagent, depending on the sensitivity of antibody and abundance of antigen. For reprobing the blot, membranes were treated with stripping buffer (Thermo Scientific) for 2–3 hrs, followed by 10% dry milk in TBST and proceeded with staining as mentioned above.

### Islet isolation from pancreas

Islets were isolated from pancreata from 2–3 month old wild type mice using the method of Szot et al. *J*. *Vis*. *Exp*. 2007 [72] with minor modifications. In brief, pancreata were perfused through common bile duct with freshly prepared Collagenase P (0.09 gm/100 ml HBSS, Roche). Inflated pancreas were subsequently removed and digested with Collagenase P at 37°C. Collagenase was removed by two washes in HBSS + 0.02% BSA. Subsequently, digested pancreata were subjected to Ficoll density gradient centrifugation (5 ml of 27%, 2.5 ml of 23%, 2.5 ml of 10.5%, 2.5 ml of 11%). Islets were hand-picked from top three layers of the gradient.

### ELISA

The ELISA assay was performed on pancreatic lysates using EZRMI-13K kit.

### Statistical Methods

All P-values were calculated by Student’s t test, comparing DMSO and SB-747651A treated samples in independent experiments, *≤0.05, **≤0.01, ***≤0.001 (Figs 3, 4, 5 and 6). For ChIP, all P-values were calculated by paired Student’s t test, comparing enrichment over IgG in independent experiments, *≤ 0.05, **≤ 0.01, ***≤0.001, one-tailed (Figs 1, 2 and 8).

## Supporting Information

S1 Fig Onecut1 staining in E15.5 pancreata, sorting and expression analysis of E12.5 *Pdx1*:*Egfp* pancreatic cells using FACS and RT-qPCR respectively. (A-C) Epifluorescence imaging of Oc1 (red) and Insulin1/2 in pancreatic sections from E15.5 stage. (D) FACS Sort of EGFP cells from *Pdx1*: *Egfp* at E12.5 (n≥15 biological replicates). (E) RT-qPCR analysis of select genes in E12.5 *Pdx1*:*Egfp* positive cells relative to *Pdx1*:*Egfp* negative cells
(TIF)

S2 Fig Epifluorescent images from select experiments. (A-D) Epifluorescence images showing nuclear expression of Msk1S360ph and Msk2T586ph at low and high magnifications in pancreatic sections from E15.5 stage. (E-F) Pdx1 immunostaining on sections from pancreatic explants treated with DMSO and SB747541A from E12.5 stage for 4 days. (G-H) Pdx1 and Insulin1/2 coimmunostaining on sections from pancreatic explants from E15.5 stage treated with DMSO and SB747541A for 4 days. (I-L) Images showing low cell density cultures of live *Pdx1*:*Egfp* medium and *Pdx1*:*Egfp* high cells treated with SB747541A after one day of culture. Arrows show that the sorted *Pdx1*:*Egfp+* pancreatic cells are in minimal contact with each other. (M-T) Insulin1/2 immunostaining on sections from pancreatic explants treated with DMSO and SB747541A from E15.5 stage, for 4 days and the corresponding brightfield images (Q-T).
(TIF)

S3 Fig **Western blot and quantification of H3S28ph and H3S10ph levels in pancreas from the indicated genotype** (A-D).
(TIF)

S4 Fig Expression analysis of the indicated genes in the dmso and SB747541A treated pancreatic explants at different developmental stages. (A, B) Expression of indicated genes in dmso treated controls at day1 (grey) and day4 (blue) of culture, by RT-qPCR. (C, D) Immunohistochemistry for Insulin1/2 on pancreatic sections from E12.5 explants cultured for 1 (C) or 4 days (D). (E-J) RT-qPCR analysis of indicated genes in pancreatic explants from E12.5 or E15.5 stage treated with SB747541A for 4 days. This is a composite data from Figs 3B, 4B and 5A–5D. Values are a ratio of normalized expression in SB747541A and normalized expression in DMSO, two independent experiments ± standard error.
(TIF)

S5 Fig Neurog3 and Amylase co-staining on day1 of SB747541A treatment in E15.5 explants. (A-F) Immunohischemical staining, showing co-expression of Neurog3 and Amylase, on pancreatic sections from E15.5 explants cultured in DMSO or SB747541A, cultured for one day. Panels A, B, D, E show single color images of the Neurog3 (A, D) or Amylase (B, E).
(TIF)

S6 Fig Analysis of cell division and apoptosis upon SB747541A treatment at the indicated days after inhibitor treatment. (A-C) anti-BrdU staining on Day4 on explants treated with a pulse of BrdU for 8hours on day 1. Total number of BrdU positive cells normalized to total area was not significantly different between DMSO and SB747541A. (D-I) Staining and quantification of Cleaved Caspase3 (D-F) and TUNEL staining (G-I) on day 1 of Msk1/2 inhibition upon harvesting pancreas form E15.5. (J-O) Staining and quantification of Cleaved Caspase3 (J-L) and TUNEL staining (M-O) on day 4 of Msk1/2 inhibition, upon harvesting pancreas from E15.5. Areas were calculated using either the Histogram function of Adobe Photoshop program or by ImageJ.
(TIF)

S7 Fig RT-qPCR analysis of different sorted populations, from DBA; Pdx1:Egfp double facs sort, at the time of isolation and 3 days into the culture. (A) FACS scatter plots of single cell suspension from E15.5 *Pdx1*:*Egfp* pancreata treated without DBA or with DBA, as indicated. (B, C) Expression analysis by RT-qPCR of indicated genes in different populations obtained from FACS sorting at the time of isolation (B) and 3 days of culture (C). The y-axis shows relative enrichment.
(TIF)

S8 Fig Expression analysis of Gcg, Ins1/2 and Amylase in *Msk1* and *Msk2* mutants. (A-T) Representative images showing expression of Glucagon (A-E, scale bar = 50μm), Insulin (F-J, scale bar = 50μm) and Amylase (K-O, scale bar = 100μm,) and corresponding brightfield images (P-T) of Amylase positive domains in the indicated genotypes at E15.5. (U-W) Glucagon, Insulin, and Amylase positive areas normalized to total area in the indicated genotypes at E15.5. For Gcg, P-values are 0.01 for *Msk1+/-*, 3.3x10^-7^ for *Msk1-/-*, 2.2x10^-4^ for *Msk2+/-*, 0.02 for *Msk2-/-*, 2.2x10^-6^ for *Msk1+/-*; *Msk2+/-* and 3.1x10^-5^ for *Msk1-/-;Msk2-/-*. For Ins1, P-value = 1.5x10^-5^ for *Msk1+/-*. For Amyl, P-values are 3.3x10^-13^ for Msk2+/-, 0.004 for *Msk1+/-*; *Msk2+/-*, 1.7 x10^-6^ for *Msk1-/-*;*Msk2-/-*. Average number of sections/pancreas for Ins1 and Gcg respectively were 17, 9.8 (wild type), 17.83, 5.6 (Msk1+/-), 15.6, 5 (Msk1-/-), 16.3, 5 (Msk2-/-), 14.75, 6.375 (Msk2+/-), 13, 13.5 (Msk1+/-; Msk2+/-), 15.12, 6.75 (Msk1-/-; Msk2-/-). All P-values were calculated by Student’s t test, relative to wild type controls, *≤ 0.05, **≤ 0.01, ***≤0.001, unequal variance, two-tailed. Values are averages of independent experiments ± standard error unless otherwise stated
(TIF)

S9 Fig Representative example illustrating the calculation of area using ImageJ and images of Gcg, Ins1/2 and Amylase from *Msk2-/-* and *Msk1+/-; Msk2+/-* pancreata. (A-C) Representative pictures demonstrating the calculation of Insulin positive area by ImageJ. The original fluorescent images for calculating Insulin positive area is shown in panel A. Representative binary pictures, thresholded by ImageJ, demonstrating Insulin positive domain (B) and total pancreatic area of the same specimen by ImageJ (C) The image was first rendered to binary and then the numbers of particles were calculated at two different thresholds for Insulin positive area (B) and total area (C) respectively by ImageJ software. (D-F) Immunohistochemical staining of Gcg, Ins1, and Amylase2a in the pancreatic sections at E15.5 stage from the indicated genotypes.
(TIF)
