# Supplementary figures and images for: The Chromatin Modifier MSK1/2 Suppresses Endocrine Cell Fates during Mouse Pancreatic Development
Source: PLoS One. 2016 Dec 14;11(12):e0166703. doi: 10.1371/journal.pone.0166703 (PMC5156359; doi:10.1371/journal.pone.0166703)

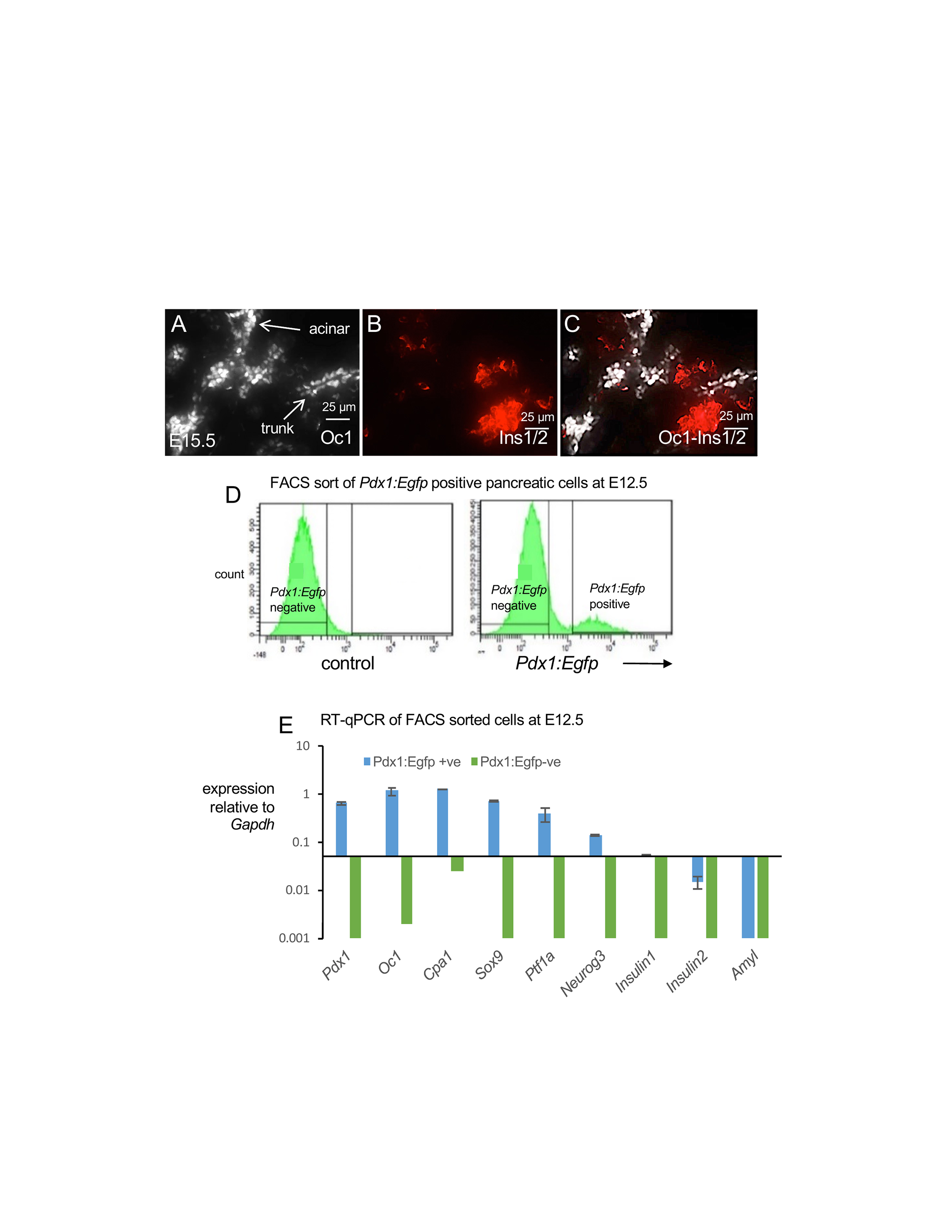

Supplement: S1 Fig — (A-C) Epifluorescence imaging of Oc1 (red) and Insulin1/2 in pancreatic sections from E15.5 stage. (D) FACS Sort of EGFP cells from Pdx1: Egfp at E12.5 (n≥15 biological replicates). (E) RT-qPCR analysis of select genes in E12.5 Pdx1:Egfp positive cells relative to Pdx1:Egfp negative cells (TIF) [file pone.0166703.s001.tif]

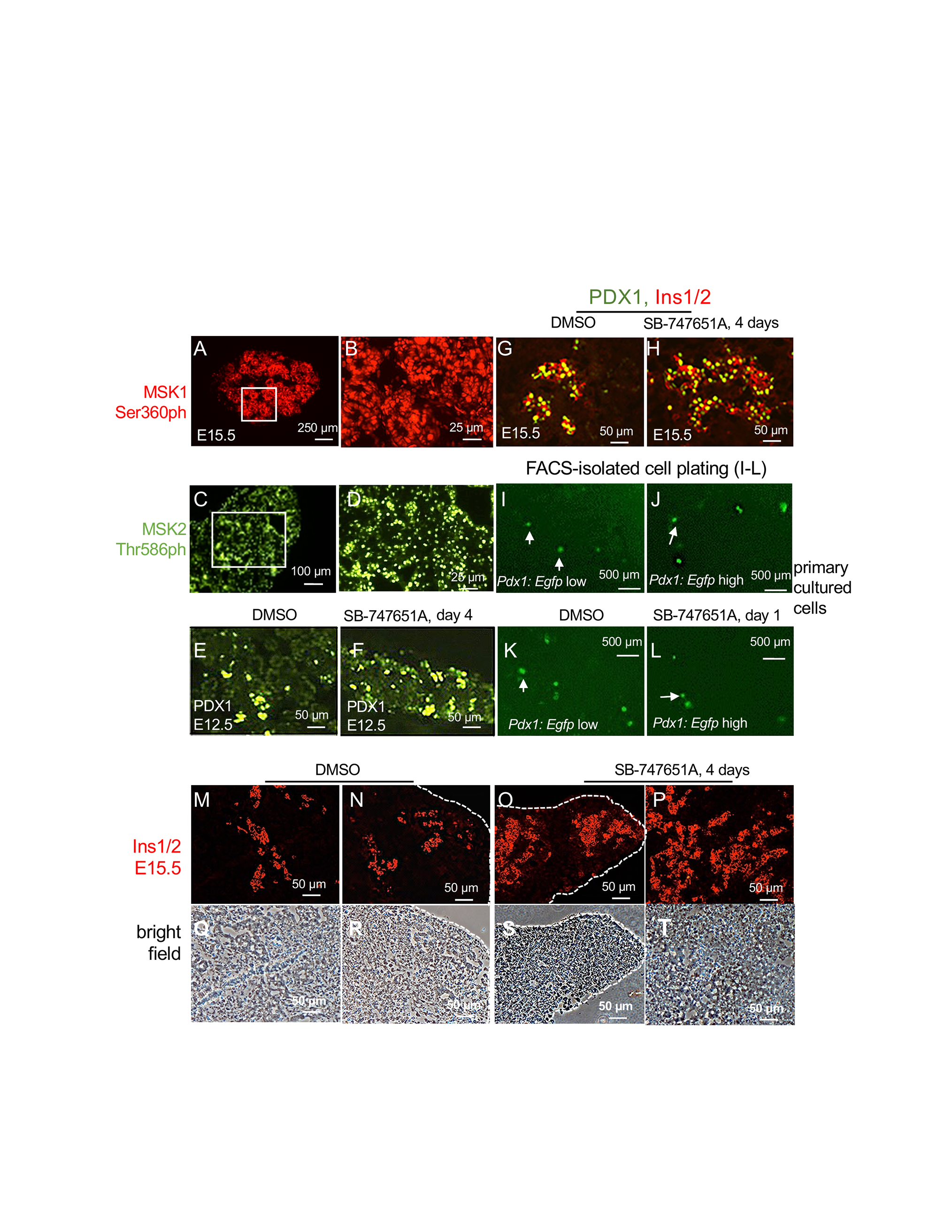

Supplement: S2 Fig — (A-D) Epifluorescence images showing nuclear expression of Msk1S360ph and Msk2T586ph at low and high magnifications in pancreatic sections from E15.5 stage. (E-F) Pdx1 immunostaining on sections from pancreatic explants treated with DMSO and SB747541A from E12.5 stage for 4 days. (G-H) Pdx1 and Insulin1/2 coimmunostaining on sections from pancreatic explants from E15.5 stage treated with DMSO and SB747541A for 4 days. (I-L) Images showing low cell density cultures of live Pdx1:Egfp medium and Pdx1:Egfp high cells treated with SB747541A after one day of culture. Arrows show that the sorted Pdx1:Egfp+ pancreatic cells are in minimal contact with each other. (M-T) Insulin1/2 immunostaining on sections from pancreatic explants treated with DMSO and SB747541A from E15.5 stage, for 4 days and the corresponding brightfield images (Q-T). (TIF) [file pone.0166703.s002.tif]

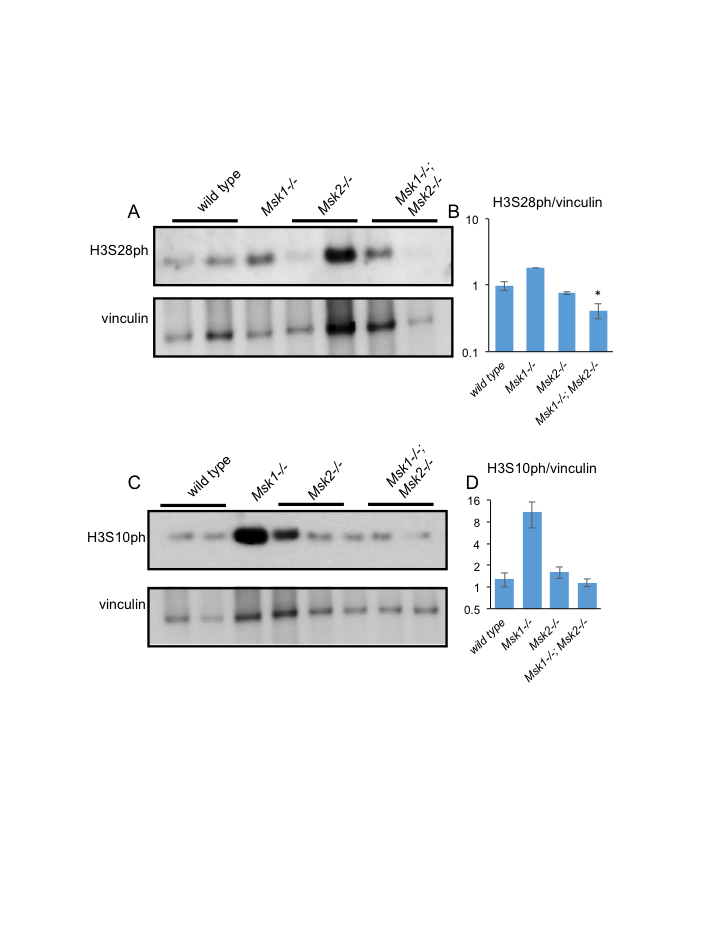

Supplement: S3 Fig — Western blot and quantification of H3S28ph and H3S10ph levels in pancreas from the indicated genotype (A-D). (TIF) [file pone.0166703.s003.tif]

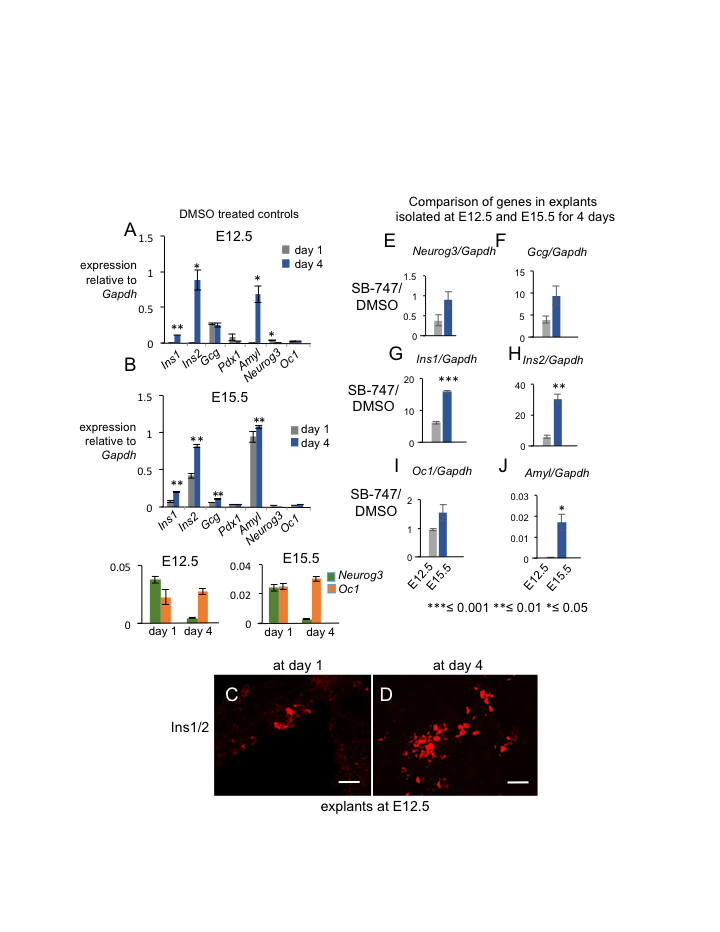

Supplement: S4 Fig — (A, B) Expression of indicated genes in dmso treated controls at day1 (grey) and day4 (blue) of culture, by RT-qPCR. (C, D) Immunohistochemistry for Insulin1/2 on pancreatic sections from E12.5 explants cultured for 1 (C) or 4 days (D). (E-J) RT-qPCR analysis of indicated genes in pancreatic explants from E12.5 or E15.5 stage treated with SB747541A for 4 days. This is a composite data from Figs 3B, 4B and 5A–5D. Values are a ratio of normalized expression in SB747541A and normalized expression in DMSO, two independent experiments ± standard error. (TIF) [file pone.0166703.s004.tif]

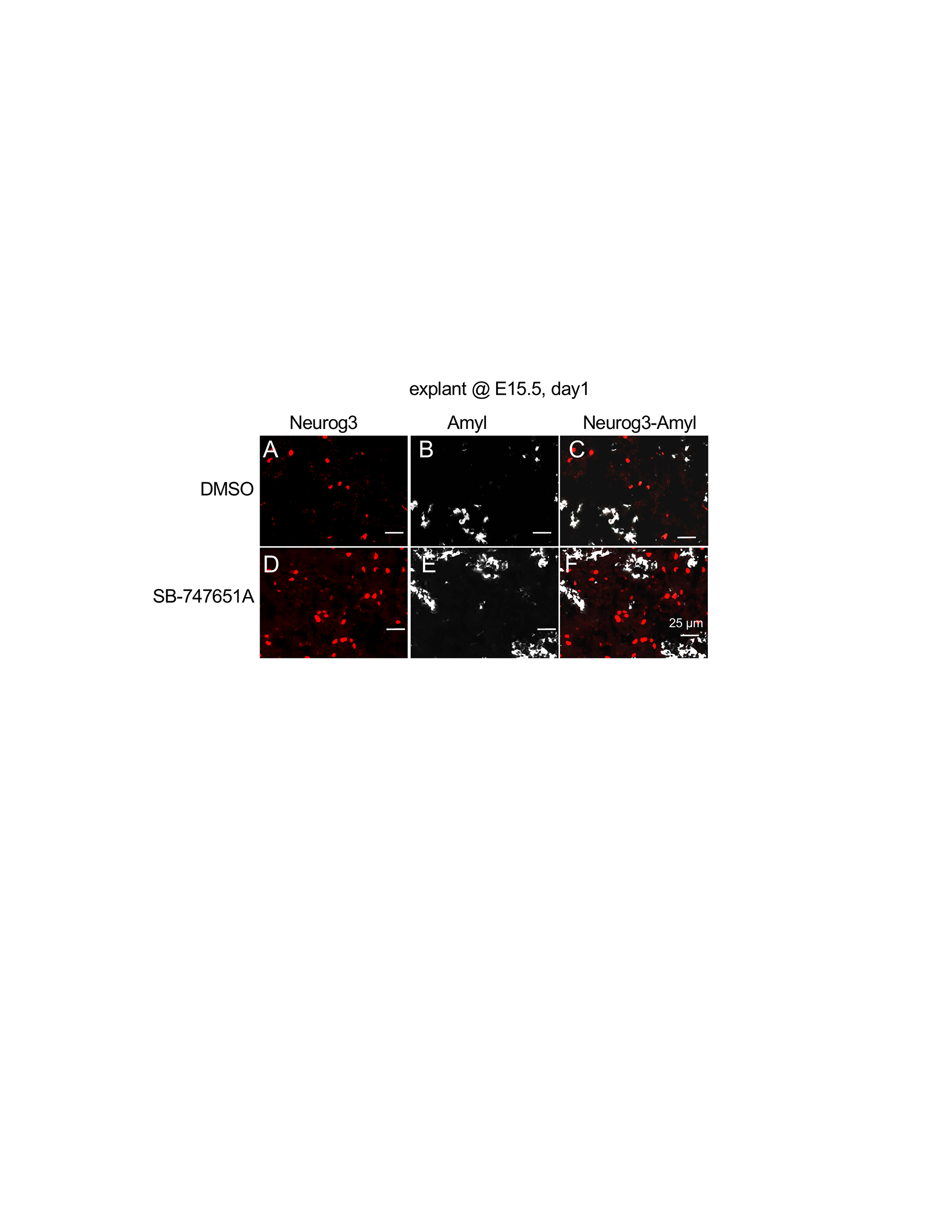

Supplement: S5 Fig — (A-F) Immunohischemical staining, showing co-expression of Neurog3 and Amylase, on pancreatic sections from E15.5 explants cultured in DMSO or SB747541A, cultured for one day. Panels A, B, D, E show single color images of the Neurog3 (A, D) or Amylase (B, E). (TIF) [file pone.0166703.s005.tif]

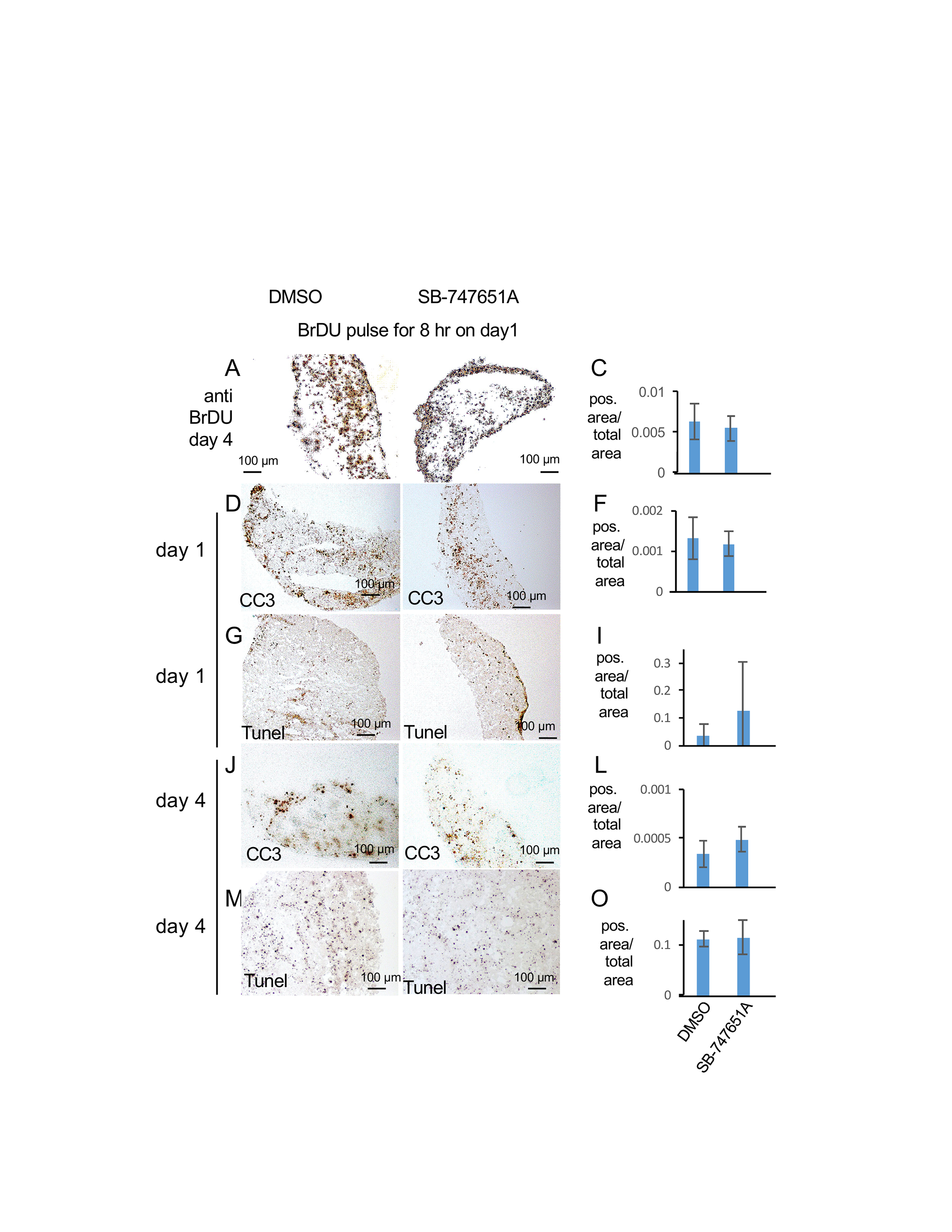

Supplement: S6 Fig — (A-C) anti-BrdU staining on Day4 on explants treated with a pulse of BrdU for 8hours on day 1. Total number of BrdU positive cells normalized to total area was not significantly different between DMSO and SB747541A. (D-I) Staining and quantification of Cleaved Caspase3 (D-F) and TUNEL staining (G-I) on day 1 of Msk1/2 inhibition upon harvesting pancreas form E15.5. (J-O) Staining and quantification of Cleaved Caspase3 (J-L) and TUNEL staining (M-O) on day 4 of Msk1/2 inhibition, upon harvesting pancreas from E15.5. Areas were calculated using either the Histogram function of Adobe Photoshop program or by ImageJ. (TIF) [file pone.0166703.s006.tif]

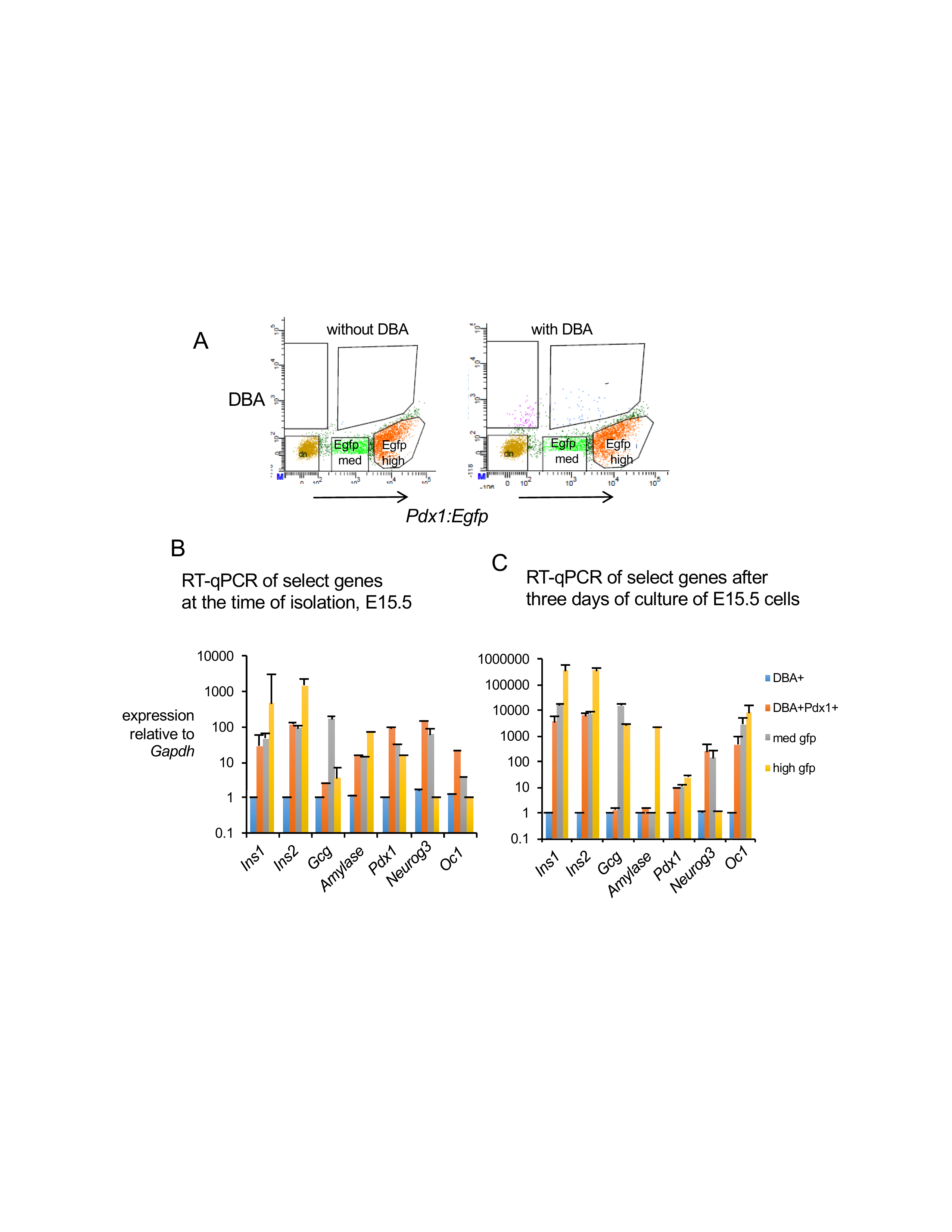

Supplement: S7 Fig — (A) FACS scatter plots of single cell suspension from E15.5 Pdx1:Egfp pancreata treated without DBA or with DBA, as indicated. (B, C) Expression analysis by RT-qPCR of indicated genes in different populations obtained from FACS sorting at the time of isolation (B) and 3 days of culture (C). The y-axis shows relative enrichment. (TIF) [file pone.0166703.s007.tif]

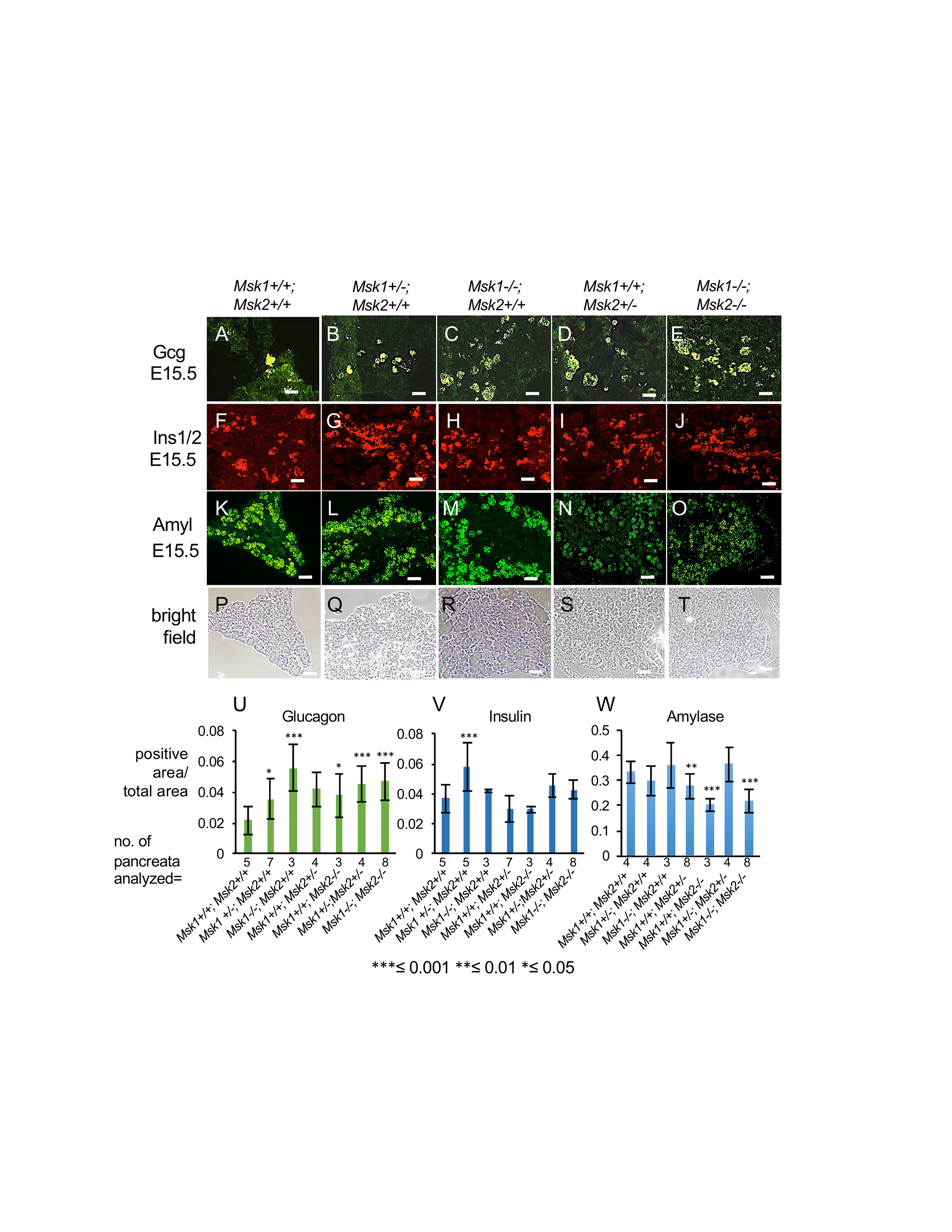

Supplement: S8 Fig — (A-T) Representative images showing expression of Glucagon (A-E, scale bar = 50μm), Insulin (F-J, scale bar = 50μm) and Amylase (K-O, scale bar = 100μm,) and corresponding brightfield images (P-T) of Amylase positive domains in the indicated genotypes at E15.5. (U-W) Glucagon, Insulin, and Amylase positive areas normalized to total area in the indicated genotypes at E15.5. For Gcg, P-values are 0.01 for Msk1+/-, 3.3x10^-7^ for Msk1-/-, 2.2x10^-4^ for Msk2+/-, 0.02 for Msk2-/-, 2.2x10^-6^ for Msk1+/-; Msk2+/- and 3.1x10^-5^ for Msk1-/-;Msk2-/-. For Ins1, P-value = 1.5x10^-5^ for Msk1+/-. For Amyl, P-values are 3.3x10^-13^ for Msk2+/-, 0.004 for Msk1+/-; Msk2+/-, 1.7 x10^-6^ for Msk1-/-;Msk2-/-. Average number of sections/pancreas for Ins1 and Gcg respectively were 17, 9.8 (wild type), 17.83, 5.6 (Msk1+/-), 15.6, 5 (Msk1-/-), 16.3, 5 (Msk2-/-), 14.75, 6.375 (Msk2+/-), 13, 13.5 (Msk1+/-; Msk2+/-), 15.12, 6.75 (Msk1-/-; Msk2-/-). All P-values were calculated by Student’s t test, relative to wild type controls, *≤ 0.05, **≤ 0.01, ***≤0.001, unequal variance, two-tailed. Values are averages of independent experiments ± standard error unless otherwise stated (TIF) [file pone.0166703.s008.tif]

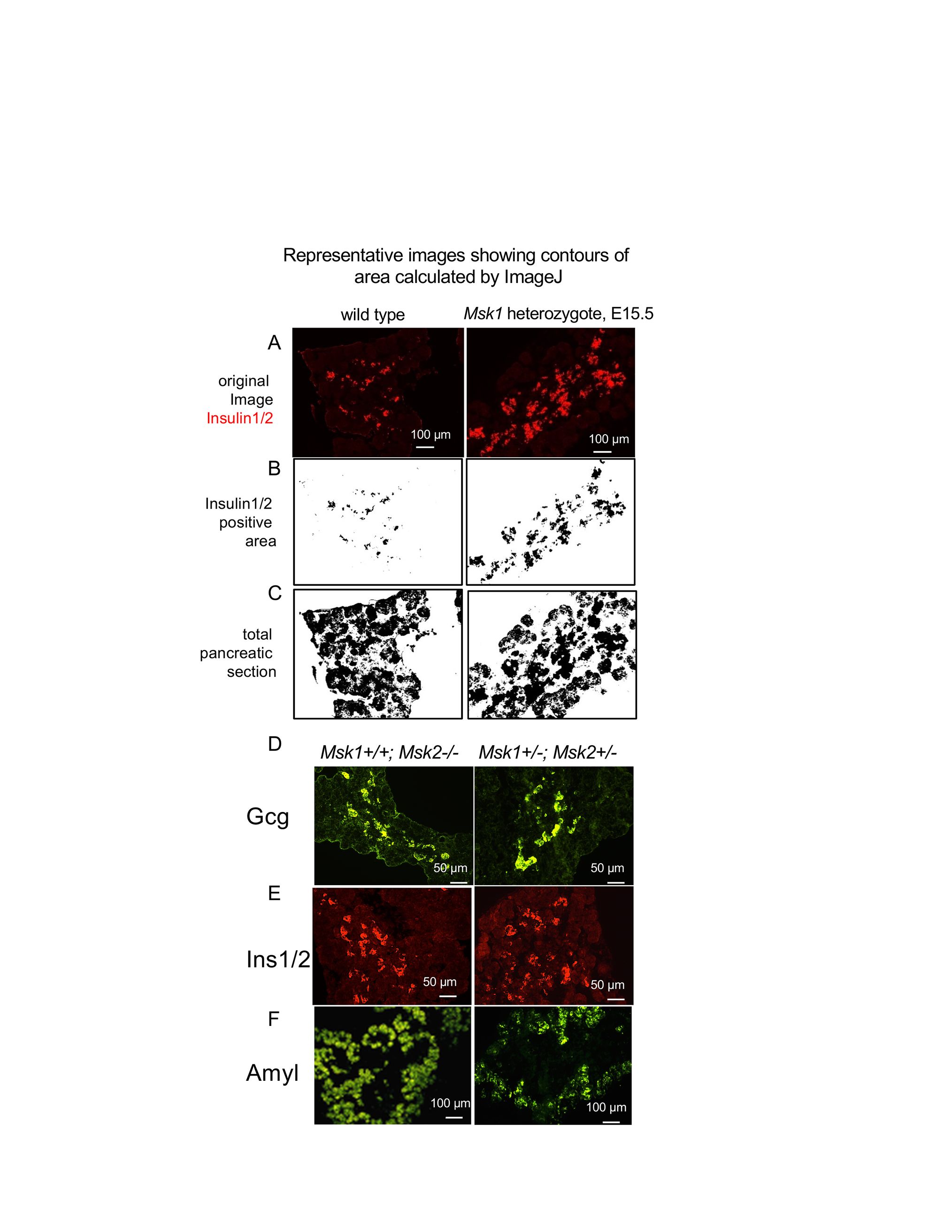

Supplement: S9 Fig — (A-C) Representative pictures demonstrating the calculation of Insulin positive area by ImageJ. The original fluorescent images for calculating Insulin positive area is shown in panel A. Representative binary pictures, thresholded by ImageJ, demonstrating Insulin positive domain (B) and total pancreatic area of the same specimen by ImageJ (C) The image was first rendered to binary and then the numbers of particles were calculated at two different thresholds for Insulin positive area (B) and total area (C) respectively by ImageJ software. (D-F) Immunohistochemical staining of Gcg, Ins1, and Amylase2a in the pancreatic sections at E15.5 stage from the indicated genotypes. (TIF) [file pone.0166703.s009.tif]
